# 6-bromo-indirubin-3′-oxime (6BIO), a Glycogen synthase kinase-3β inhibitor, activates cytoprotective cellular modules and suppresses cellular senescence-mediated biomolecular damage in human fibroblasts

**DOI:** 10.1038/s41598-017-11662-7

**Published:** 2017-09-15

**Authors:** Aimilia D. Sklirou, Nicolas Gaboriaud-Kolar, Issidora Papassideri, Alexios-Leandros Skaltsounis, Ioannis P. Trougakos

**Affiliations:** 1 Department of Cell Biology and Biophysics, Faculty of Biology, National and Kapodistrian University of Athens, Athens, 15784 Greece; 2 Department of Pharmacognosy and Natural Products Chemistry, Faculty of Pharmacy, National and Kapodistrian University of Athens, Athens, 15771 Greece

## Abstract

As genetic interventions or extended caloric restriction cannot be applied in humans, many studies have been devoted to the identification of natural products that can prolong healthspan. 6-bromoindirubin-3′-oxime (6BIO), a hemi-synthetic derivative of indirubins found in edible mollusks and plants, is a potent inhibitor of Glycogen synthase kinase 3β (Gsk-3β). This pleiotropic kinase has been implicated in various age-related diseases including tumorigenesis, neurodegeneration and diabetes. Accordingly, 6BIO has shown anti-tumor and anti-neurodegenerative activities; nevertheless, the potential role of 6BIO in normal human cells senescence remains largely unknown. We report herein that treatment of human diploid skin fibroblasts with 6BIO reduced the oxidative load, conferred protection against oxidative stress-mediated DNA damage, and it also promoted the activation of antioxidant and proteostatic modules; these effects were largely phenocopied by genetic inhibition of Gsk-3. Furthermore, prolonged treatment of cells with 6BIO, although it decreased the rate of cell cycling, it significantly suppressed cellular senescence-related accumulation of biomolecular damage. Taken together, our presented findings suggest that 6BIO is a novel activator of antioxidant responses and of the proteostasis network in normal human cells; moreover, and given the low levels of biomolecules damage in 6BIO treated senescing cells, this compound likely exerts anti-tumor properties.

## Introduction

Organismal ageing is an inevitable and irreversible consequence of life promoted by both genetic and environmental factors^1,2^. Specifically, ageing is defined as a time-dependent decline of stress resistance and functional capacity, associated with increased probability of morbidity and mortality. These effects relate to (among others) age-related gradual accumulation of damaged biomolecules (including proteins) which eventually result in the disruption of cellular homeodynamics. Accordingly, ageing is the primary risk factor for major human pathologies, including cancer, diabetes, cardiovascular disorders and neurodegenerative diseases^2^.

Proteome quality control is critical for cellular functionality and it is assured by the curating activity of the proteostasis network (PN) and of antioxidant responses. Central to PN functionality are the two main proteolytic systems, namely the Ubiquitin-Proteasome System (UPS) and the Autophagy-Lysosome Pathway (ALP) along with the armada of the molecular chaperones^3,4^. UPS degrades both normal short-lived ubiquitinated proteins, as well as non-repairable misfolded, unfolded or damaged proteins^3,5,6^, whereas ALP is mostly involved in the degradation of long-lived proteins, aggregated ubiquitinated proteins and in the recycling of damaged organelles^7–9^. On the other hand, molecular chaperones are mostly responsible for the correct folding of proteins and for the prevention of protein aggregation. Moreover, they either refold unfolded and misfolded proteins or drive them for degradation through the two aforementioned degradation machineries^10,11^. Proteome quality control also depends on the activity of the Nrf2 (Nuclear factor erythroid 2-related factor 2)/Keap1 (Kelch-like ECH-associated protein 1) signalling pathway which regulates cellular responses to oxidative and electrophilic stress. Nrf2 is a key transcription factor regulating the expression of a wide array of phase II and antioxidant enzymes; under normal conditions Nrf2 is inhibited in the cytoplasm by Keap1^12^.

The UPS functionality, as well as the antioxidant responses signalling, decline during cellular senescence or *in vivo* ageing^13–17^ indicating that they are involved in the appearance and, likely, the progression of ageing phenotypes. On the other hand, activation of UPS and of stress responsive pathways has been linked to prolonged efficient removal of damaged and/or dysfunctional polypeptides, exerting thus anti-ageing effects^18–21^.

It is nowadays evident that both healthspan (the disease-free period of life) and/or lifespan (maximum longevity) can be prolonged by genetic, dietary (e.g. caloric restriction) and/or pharmacological interventions suggesting that animals have the latent potential to live longer than they normally do^1,2,22^. As genetic interventions or prolonged caloric restriction cannot be applied in humans, many studies have been devoted to the identification of natural products (NPs) that can prolong healthspan and/or lifespan. It is well established that NPs represent an extraordinary inventory of high diversity structural scaffolds that can be used as pharmacological modulators of age-related signalling pathways. These pathways may be involved in ageing regulation by dampening signalling from nutrient sensing pathways, thus mimicking the systemic effects of caloric restriction or by activating the stress responsive pathways^1^. Nevertheless, and despite encouraging findings in relation of NPs potential bioactivity towards the delay of cellular senescence and/or *in viv*o ageing, the greatest part of world’s plant, marine or microbial bio- and chemo-diversity is un-investigated; furthermore in most cases the mechanism of NPs action remains elusive.

Given these facts we are currently performing an extensive high-throughput screening of thousands different NPs (e.g. crude extracts, microfractions or purified compounds) originating from different sources of the biosphere (e.g. marine organisms, plants and microorganisms) in order to identify bioactive molecules against age-related proteome instability, cellular senescence and *in vivo* ageing; these data along with the targets and bioactive lead molecules will be reported elsewhere.

Our herein presented study was focused on bioactive indirubins and specifically a hemi-synthetic cell-permeable indirubin derivative, namely 6-bromoindirubin-3′-oxime (6BIO). Indirubins belong to the family of bis-indole alkaloids isolated from indigo dye-producing edible plants and gastropod mollusks^23^. Indirubins and their analogues have been described as potent inhibitors of Cyclin-dependent kinases (CDKs)^24^, as well as of Glycogen synthase kinase-3 β (Gsk-3β)^25^. Gsk-3 is a multifunctional ubiquitously expressed serine/threonine kinase that has been functionally involved in diverse cellular processes, including (among others) glycogen synthesis, proliferation, development and apoptosis^26–28^. In mammalian tissues, Gsk-3 exists as two isoforms (Gsk-3α and Gsk-3β) that share 98% homology of their kinase domains, while differing substantially in their N-terminal and C-terminal sequences^29^. Accordingly, Gsk-3 has been involved in several age-related conditions and diseases including inflammation, diabetes, neurodegenerative disorders and cancer^28,30–32^. Nevertheless, the role of either Gsk-3β or 6BIO in normal human cells senescence remains largely unknown. We report herein that 6BIO is a novel modulator of antioxidant responses and PN activity in human fibroblasts. Moreover, prolonged incubation of cells with 6BIO decreased the rate of cell cycling and resulted in cells that senesce in the absence of a major cellular senescence hallmark, namely biomolecular damage.

## Results

### At relatively low concentrations (up to 5 μM) 6BIO was not cytotoxic in normal human fibroblasts; it reduced cellular oxidative load and conferred protection against oxidative or genotoxic stress

**Figure 1:**
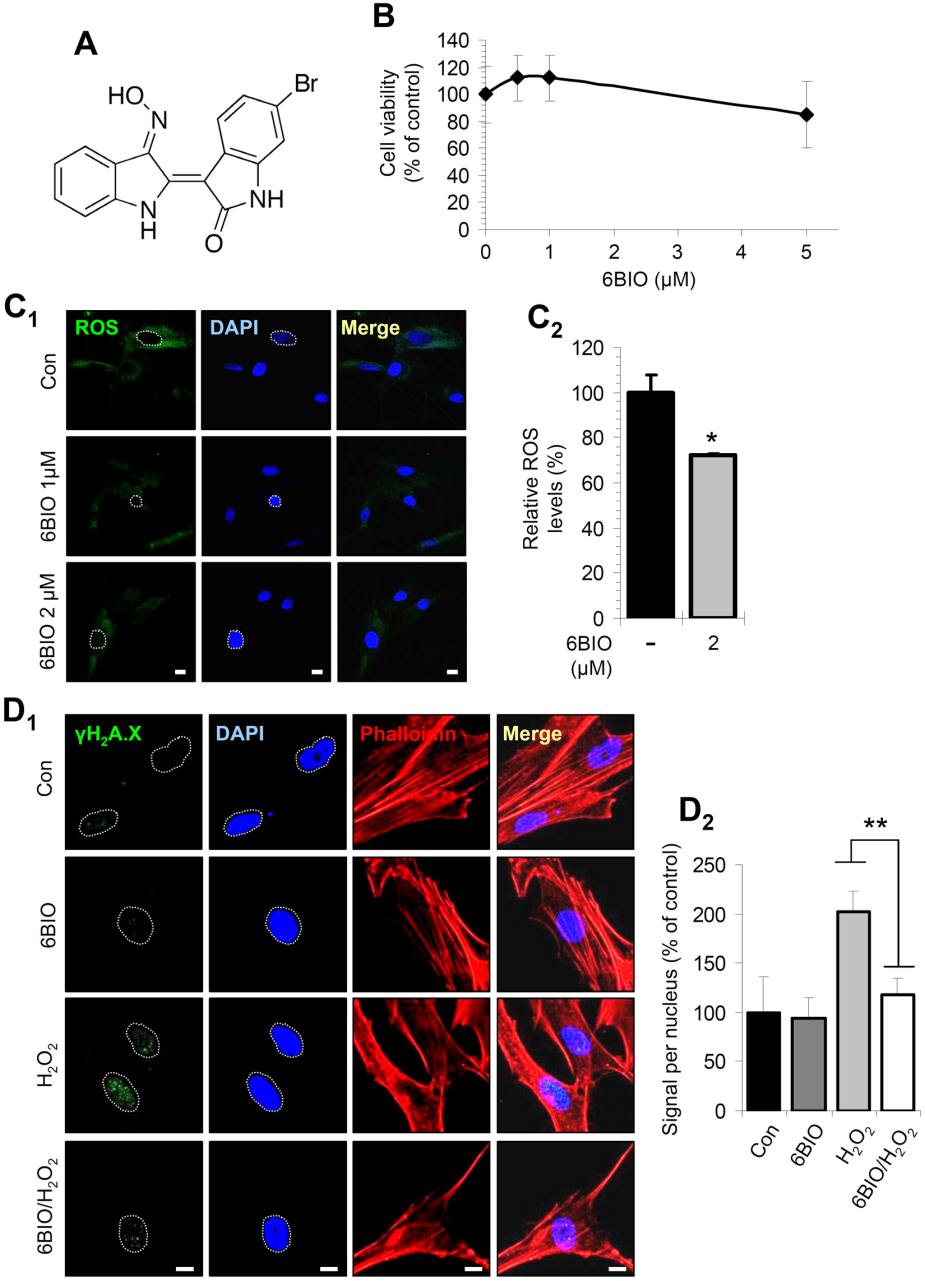
At concentrations up to 5 μM 6BIO exerted no toxicity in human skin fibroblasts; it reduced cellular oxidative load and conferred protection against oxidative stress-mediated DNA damage. (**A**) 6BIO chemical structure. (**B**) Relative (%) survival (MTT assay) of BJ fibroblasts exposed to the indicated concentrations of 6BIO for 24 h. (**C**_1_) ROS levels in living cells treated with the shown concentrations of 6BIO for 24 h; ROS were stained with the CM-H_2_DCFDA dye and viewed by CLSM. (**C**_2_) Relative ROS levels after cell treatment for 24 h with 2 μM of 6BIO; shown (%) values represent fluorometry measurement of ROS in live cells by microplate analysis. (**D**_1_) Representative CLSM images following immunofluorescence staining of the Ser^139^ phosphorylated form of histone H_2_A.X (γH_2_A.X); cells were treated for 24 h with 200 μM H_2_O_2_ in the presence or absence of 2 μM 6BIO. (**D**_2_) Relative quantification of the γH_2_A.X foci staining intensity per nucleus. In (**C**_1_,**D**_1_) cells nuclei were counterstained with DAPI (blue) and actin with Phalloidin (red). Control samples values were set to 100%. Bars, ±SD; *P < 0.05; **P < 0.01. Bars, in (**C**_1_,**D**_1_) 10 μM.

Firstly, we examined the effect of 6BIO (Fig. 1A) on the viability of BJ skin fibroblasts. To this end, cells were incubated with increasing concentrations of 6BIO for 24 h and we found that at low concentrations (up to 5 μM) 6BIO exerted no significant toxicity to human fibroblasts (Fig. 1B); yet, at higher concentrations 6BIO was toxic as it reduced cell viability by ~50% (Suppl. Fig. S1). Then, we investigated whether 6BIO affects the levels of cellular Reactive Oxygen Species (ROS). Cells were treated with 6BIO for 24 h and the intracellular ROS levels were evaluated by either CLSM (Fig. 1C1) or by fluorometry (Fig. 1C2); both assays revealed that 6BIO significantly decreased ROS levels in BJ cells.

We also examined if 6BIO could protect cells from the genotoxic impact of oxidants or genotoxic agents. We initially assessed the phosphorylation of H_2_A.X (termed γΗ_2_Α.Χ) on Ser^139^; a marker of DNA damage and genotoxic stress^33^. We found that co-incubation of cells with 6BIO and the oxidative agent H_2_O_2_ (200 μM) for 24 h led to the formation of less DNA damage foci in cells nuclei as compared to cells treated with only H_2_O_2_ (Fig. 1D). The protective effects of 6BIO against oxidative and/or genotoxic stress was also confirmed by immunoblotting analyses after treating BJ cells with the chemotherapeutic drug doxorubicin (DXR). Specifically, it was found that co-treatment of BJ cells with 6BIO and DXR resulted in less intense (as compared to treatment with DXR alone) p53 activation (shown by reduced p53 levels and p53 phosphorylation at Ser-15) and γΗ_2_Α.Χ phosphorylation (Suppl. Fig. S2).

### Treatment of human fibroblasts with 6BIO activated proteostatic and antioxidant modules

Given the fact that 6BIO has been isolated as a specific inhibitor of Gsk-3β^25,34^, we then studied the 6BIO-mediated effects on the *gsk-3β* gene expression, as well as on the Akt-mediated Ser^21/9^ inhibitory phosphorylation on Gsk-3. We noted that short-term treatment of BJ cells with 6BIO upregulated both the *gsk-3β* gene (Suppl. Fig. S3A) and Gsk-3β protein (Suppl. Fig. S3B; upper panel) expression levels; moreover, it suppressed the inhibitory Gsk-3 Ser^21/9^ phosphorylation (Suppl. Fig. S3B; middle panel) indicating the existence of a regulatory feedback loop which, upon 6BIO-mediated Gsk-3 inhibition, aims to restore physiological cellular levels of Gsk-3 kinase activity.

**Figure 2:**
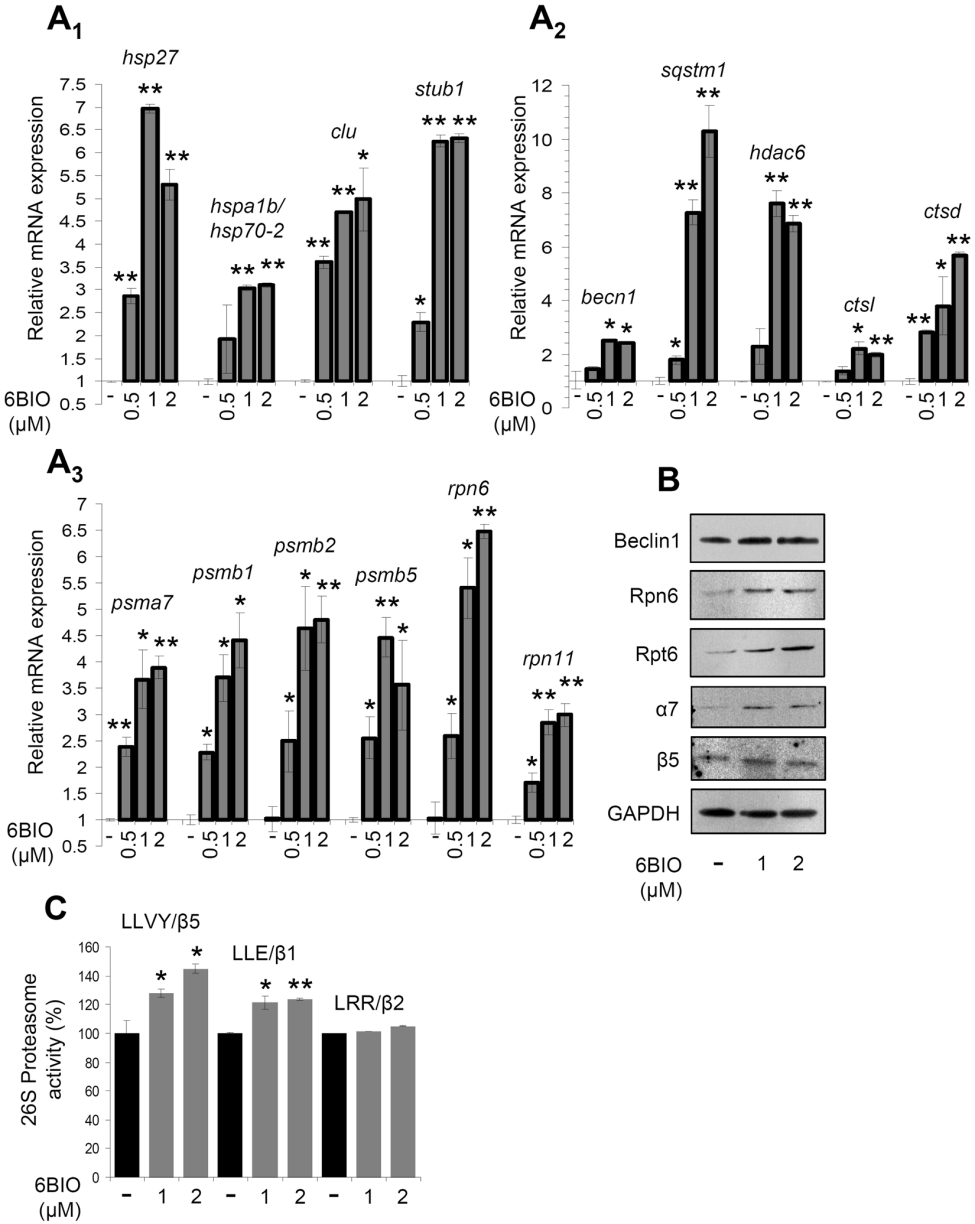
Treatment of human skin fibroblasts with 6BIO activated proteostatic modules. (**A**_1_–**A**_3_) Relative expression of molecular chaperones genes (*hsp27*, *hspa1b/hsp70-2*, *clu*, *stub1*) (**A**_1_), of genes involved in ALP functionality (*becn1*, *sqstm1*, *hdac6*, *ctsl*, *ctsd*) (**A**_2_), as well as of proteasomal genes (*psma7*, *psmb1*, *psmb2*, *psmb5*, *rpn6*, *rpn11*) (**A**_3_), in cells treated with the indicated concentrations of 6BIO for 24 h. (**B**) Representative immunoblot analyses of the autophagy-related Beclin1 and the 26S proteasome subunits Rpn6, Rpt6, α7 and β5 expression levels after cell exposure to the shown concentrations of 6BIO for 36 h. (**C**) Relative proteasome peptidase activities (LLVY/β5, LLE/β1 and LRR/β2) in cells treated with 1 or 2 μM 6BIO for 36 h. The beta-2-microglobulin (*b2m*) gene expression (**A**_1_–**A**_3_) and GAPDH probing (**B**) were used as reference for RNA and protein input, respectively. In (**A**_1_–**A**_3_) control samples values were set to 1; in (**C**) control values were set to 100%. Quantitation of shown blots is presented in Suppl. Fig. S10. Bars, ± SD; *P < 0.05; **P < 0.01.

**Figure 3:**
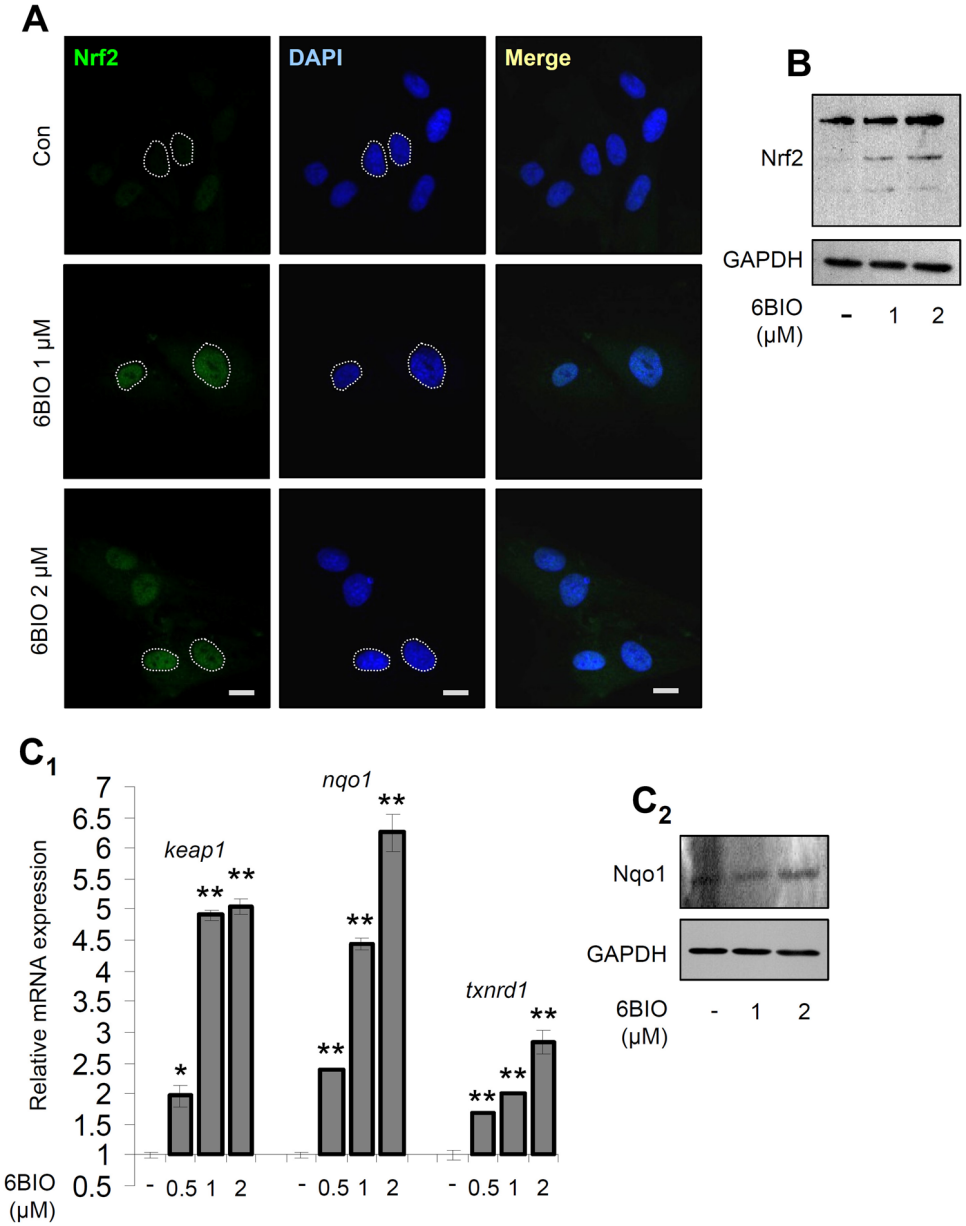
6BIO administration in human skin fibroblasts enhanced Nrf2 nuclear accumulation and upregulated Nrf2 transcriptional targets. (**A**) CLSM images following Nrf2 immunofluorescence localisation in cells exposed to the shown concentrations of 6BIO for 24 h; cells nuclei were counterstained with DAPI. (**B**) Representative immunoblotting analysis of Nrf2 expression (shown bands represent different Nrf2 forms^66^) in fibroblasts treated with 1 or 2 μM 6BIO for 36 h. (**C**_1_) Relative expression of Nrf2 transcriptional target genes (*keap1*, *nqo1*, *txnrd1*) after exposing cells to the indicated concentrations of 6BIO for 24 h. (**C**_2_) Immunoblotting analysis of Nqo1 expression after cell exposure (or not) to the shown concentrations of 6BIO for 36 h. Probing with GAPDH was used as total protein loading reference in (**B**,**C**_2_); quantitation of shown blots is presented in Suppl. Fig. S10. The *b2m* gene expression (**C**_1_) was used as normaliser. Bars, ±SD; *P < 0.05; **P < 0.01 *vs*. controls set to 1. Bars, in (**A**) 10 μM.

We then investigated the effect of 6BIO on cellular antioxidant and proteostatic modules. We observed that short-term (24 h) cell exposure to 6BIO induced a dose-dependent upregulation of molecular chaperones genes (*hsp27*, *hspa1b/hsp70-2*, *clu*, *stub1*) (Fig. 2A1); of genes involved in ALP functionality (*becn1*, *sqstm1*, *hdac6*, *ctsl*, *ctsd*) (Fig. 2A2), as well as of 20S (*psma7*, *psmb1*, *psmb2*, *psmb5*) and 19S (*rpn6*, *rpn11*) proteasomal genes (Fig. 2A3). In line with these findings, we noted mild upregulation of the autophagy-related protein Beclin1 and of the proteasomal subunits Rpn6, Rpt6, α7 and β5 (Fig. 2B). Furthermore, cell exposure to 6BIO resulted in increased proteasomal peptidase activities (Fig. 2C). Given the fact that Gsk-3 has been previously involved in suppressing Nrf2 activity^35,36^, we asked whether part of these 6BIO induced pleiotropic effects are Nrf2-mediated. Immunofluorescence analyses showed that short-term exposure of BJ cells to 6BIO enhanced the nuclear accumulation of the transcription factor Nrf2 (Fig. 3A); it also resulted in higher Nrf2 protein expression levels (Fig. 3B), apparently due to Nrf2 activation and stabilisation. In accordance with these findings, we noted that cell treatment with 6BIO resulted in a dose-dependent upregulation of well known Nrf2 transcriptional targets, namely the *keap1*, *nqo1* and *txnrd1* genes (Fig. 3C1), as well as in mild upregulation of the Nqo1 protein (Fig. 3C2).

### Genetic inhibition of Gsk-3 largely phenocopied the 6BIO-mediated effects

**Figure 4:**
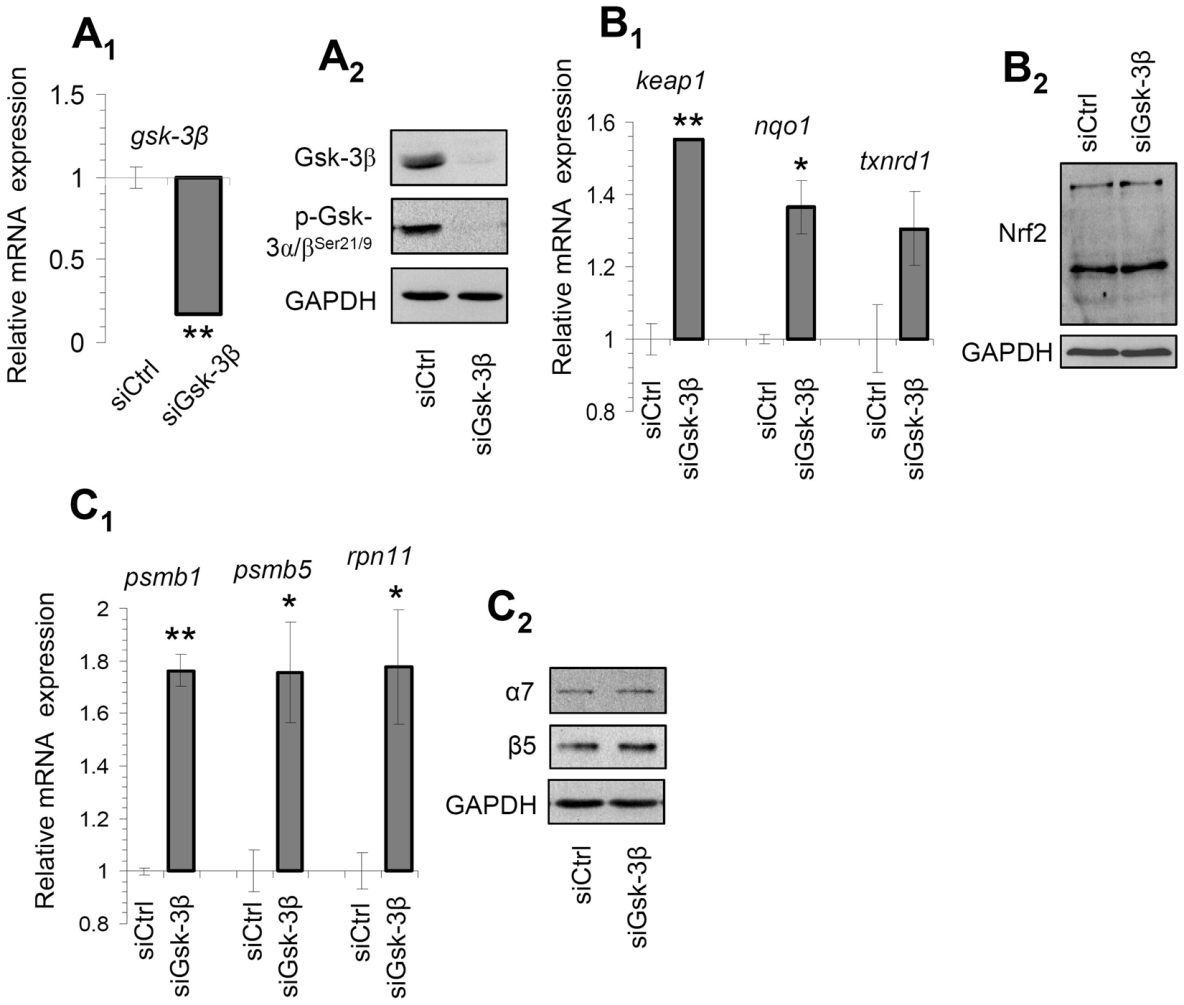
siRNA-mediated *gsk-3β* gene expression knock down in human fibroblasts largely phenocopied the 6BIO-induced effects. (**A**_1_) Q-RT-PCR assay of *gsk-3β* gene expression levels after transfecting (or not) cells with Gsk-3β RNAi oligonucleotides for 24 h. (**A**_2_) Representative immunoblot analyses of protein samples probed with antibodies against Gsk-3β and p-Gsk-3^Ser21/9^ after Gsk-3β RNAi for 48 h. (**B**_1_) Q-PCR expression analyses of Nrf2 transcriptional target genes (*keap1*, *nqo1*, *txnrd1*) after Gsk-3β RNAi for 24 h. (**B**_2_) Immunoblotting analysis of Nrf2 expression after Gsk-3β RNAi for 24 h. (**C**_1_) Relative expression of 20S (*psmb1*, *psmb5*) or 19S (*rpn11*) proteasome genes after Gsk-3β RNAi for 24 h. (**C**_2_) Representative immunoblot analyses of protein samples probed with antibodies against the 20S proteasome subunits α7 and β5 after Gsk-3β RNAi for 48 h. Probing with GAPDH was used in (**A**_2_,**B**_2_,**C**_2_) as total protein loading reference; *b2m* gene expression (**A**_1_,**B**_1_,**C**_1_) was used as reference for RNA input. Quantitation of shown blots is presented in Suppl. Fig. S10. Bars, ± SD; * P < 0.05; ** P < 0.01 *vs*. controls set to 1.

We then sought to phenocopy the 6BIO-mediated effects by applying RNAi-mediated silencing of the *gsk-3β* gene expression. We noted that effective knock down of the *gsk-3β* gene (Fig. 4A1) and protein (Fig. 4A2; upper panel) expression decreased the levels of the inhibitory Gsk-3 Ser^21/9^ phosphorylation (Fig. 4A2; middle panel). Furthermore, it resulted in upregulation of Nrf2 transcriptional targets, namely the *keap1*, *nqo1* and *txnrd1* genes (Fig. 4B1), likely as a result of increased expression (Fig. 4B2) and transcriptional activation of Nrf2. As in the case of 6BIO treatment, we also found that silencing of the *gsk-3β* gene expression promoted the induction of proteasomal (*psmb1*, *psmb5*, *rpn11*) genes (Fig. 4C1) and protein subunits (Fig. 4C2) expression. In line with these findings (and the 6BIO-mediated protection against oxidative stress-induced genotoxicity), we also noted that RNAi-mediated Gsk-3 knock down protected BJ cells from oxidative stress-mediated DNA damage (Suppl. Fig. S4).

### Long-term incubation of fibroblasts with 6BIO altered cell morphology; it reduced migratory activity and slowed down cell cycling

**Figure 5:**
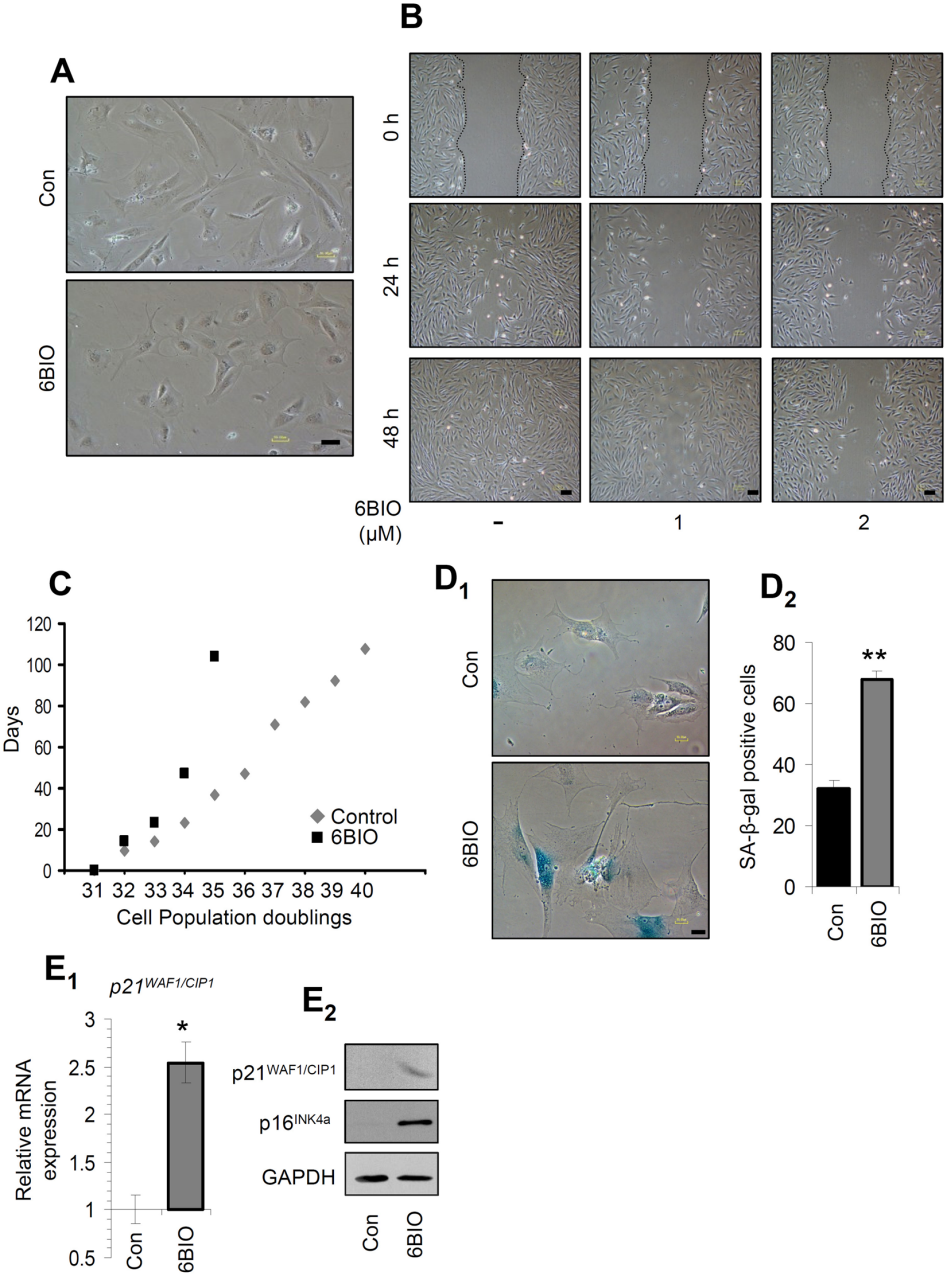
Incubation of BJ cells with 6BIO altered cell morphology; it reduced migratory activity and slowed down the rate of cell cycling. (**A**) Representative phase contrast images of BJ cells following incubation for 20 days with 6BIO. (**B**) Phase contrast images showing cellular migratory activity 24–48 h after a scratch assay in confluent BJ cell cultures; cells were treated (or not) with 1 or 2 μM 6BIO as indicated. (**C**) Cell Population Doublings performed by control cells or cells treated with 1 μM 6BIO. (**D**) Representative images (**D**_1_) and quantitation (**D**_2_) of *SA-β-Gal* staining after long-term treatment of BJ cells with 1 μM 6BIO. (**E**) *p21* ^*CIP1/WAF1*^ gene (**E**_1_) and p21^CIP1/WAF1^, p16^INK4a^ CDKIs protein (**E**_2_) expression levels in BJ cells after long-term treatment with 1 μM 6BIO. The *b2m* gene expression (**E**_1_) and GAPDH probing (**E**_2_) were used as reference for RNA and protein input, respectively. Quantitation of shown blots is presented in Suppl. Fig. S11. Bars, ±SD; *P < 0.05; **P < 0.01 *vs*. controls set to 1. Bars, in (**A**,**B**,**D**_1_) 50 μM.

Considering the findings after short-term cell exposure to 6BIO, we then investigated its long-term effects on fibroblasts. BJ cells treated for a period of 20 days with 6BIO displayed a different (compared to controls) morphology as instead of their typical spindle shape they tend to adopt an epithelial-like morphology (Fig. 5A). Moreover, we found that 6BIO delayed migratory activity of cells at an *in vitro* scratch assay (Fig. 5B), while in accordance to the reported inhibitory effects of indirubins on CDKs^24^, 6BIO treated cells were proliferating with a significant lower rate and carried out less number of population doublings as compared to control cells (Fig. 5C). Furthermore, 6BIO treated cells stained positively for the senescence specific *SA-β-Gal* activity (Fig. 5D). In support, we noted that long-term treatment of BJ cells with 6BIO promoted the upregulation of CDK inhibitors (CDKIs), namely the *p21* ^*CIP1/WAF1*^ gene (Fig. 5E1) and the p21^CIP1/WAF1^ and p16^INK4a^ proteins (Fig. 5E2) expression levels; it also (likely as part of the aforementioned regulatory feedback loop) induced the expression levels of the *gsk-3β* gene (Suppl. Fig. S5A) and Gsk-3β protein (Suppl. Fig. S5B). These data indicate that the pleiotropic effects of sustained 6BIO treatment also include the reduction of normal human fibroblasts replicative potential.

### In spite of the reduced rate of cell cycling, long-term treatment of normal human fibroblasts with 6BIO decreased the levels of cellular senescence-mediated oxidative and proteotoxic stress

**Figure 6:**
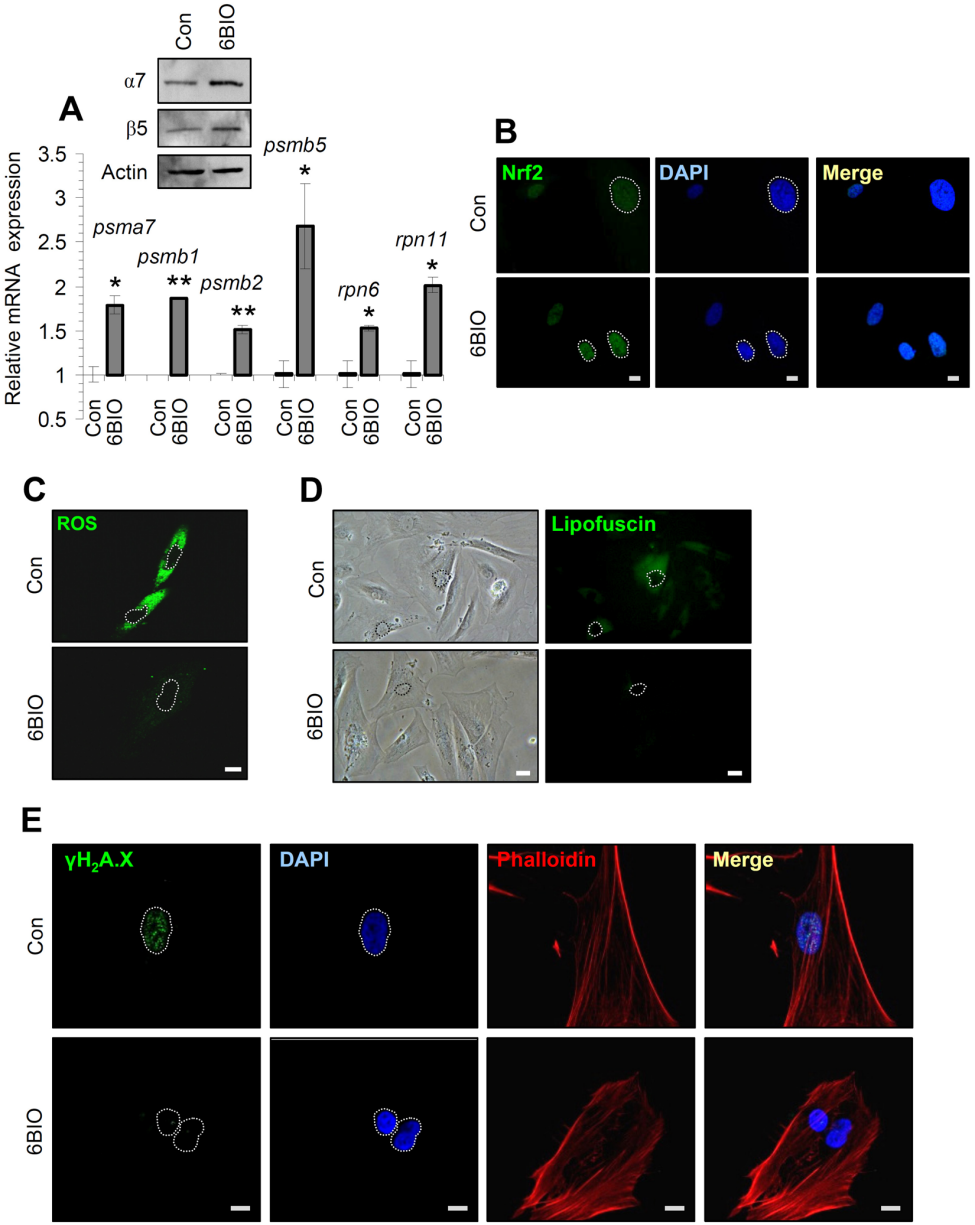
Long-term treatment of normal human fibroblasts with 6BIO significantly reduced the senescence-mediated increase in oxidative and proteotoxic stress. (**A**) Relative expression of proteasome genes (*psma7*, *psmb1*, *psmb2*, *psmb5*, *rpn6*, *rpn11*) after long-term cell treatment with 6BIO; in the upper insert the expression levels of the proteasomal subunits α7 and β5 after exposing cells to 6BIO for 25 days are shown. (**B**) Confocal analysis of cells incubated with 6BIO for 25 days after labelling with an antibody against Nrf2 and counterstaining with DAPI. (**C**) CLSM ROS detection in BJ cells after treatment with 6BIO for 25 days. (**D**) Lipofuscin fluorescence imaging after long-term treatment of BJ cells with 6BIO. (E) Representative CLSM images following detection of the Ser^139^ phosphorylated form of histone H_2_A.X (γH_2_A.X) after long-term treatment of cells with 6BIO. Cells nuclei were counterstained with DAPI (blue) and actin with Phalloidin (red). The *b2m* gene expression (**A**) was used as a total RNA loading reference. In all cases cells were treated with 1 μM 6BIO. Quantitation of shown blots is presented in Suppl. Fig. S11. Bars, ± SD; *P < 0.05; **P < 0.01 *vs*. controls set to 1. Bars, in (**B**,**C**,**E**) 10 μM, and in (**D**) 50 μM.

We then addressed the issue whether prolonged incubation of cells with 6BIO affects (as found after short-term treatment) PN and antioxidant responses-related modules. We found that long-term treatment of BJ cells with 6BIO resulted in sustained upregulation of 20S (*psma7*, *psmb1*, *psmb2*, *psmb5*) and 19S (*rpn6*, *rpn11*) proteasomal genes (Fig. 6A) and proteins (Fig. 6A; upper insert), but not anymore of genes involved in cell antioxidant responses (Suppl. Fig. S6; see also below). Moreover, long-term treatment of cells with a non cytotoxic 6BIO concentration resulted in higher rates (compared to controls) of Nrf2 accumulation in cells nuclei (Fig. 6B), as well as in significant reduction in cellular senescence-associated ROS (Fig. 6C) and lipofuscin (Fig. 6D) intracellular levels. The reduced rate of cellular senescence-associated lipofuscin accumulation was not cell type or tissue dependent, as it was also confirmed in another cell line, namely, normal human lung IMR90 fibroblasts (Suppl. Fig. S7).

To get more insight to these observations and to comparatively analyze the short- and long-term effects of 6BIO in normal human fibroblasts, we also assayed the expression levels of an extended number of genes being involved in different cellular functions, including DNA damage responses and cell survival regulation, proteome stability (i.e. chaperones, UPS and ALP), antioxidant responses, as well as mitochondrial, metabolic, hypoxia, extracellular matrix and immune responses-related pathways (Suppl. Fig. S8; see also Suppl. Materials and Methods). We found that short-term treatment of BJ cells with 6BIO resulted in a rather generalized induction of most of the studied genes (with the notable exception of *insr* and *il8*) (Suppl. Fig. S8). This effect was however largely ceased after long-term cell exposure to 6BIO, while for some genes (e.g. *puma*, *noxa*, *bax*, *hsp27*, *hspa1b*, *psmb6*, *psmb7*, *nqo1*, *txnrd1*, *pprc1*, *pdpk1*, *akt1*, *pdk1 and hif1a*) it was even tend to be inverted; indicating that cell signalling pathways retain their capacity to eventually adapt to sustained treatment with 6BIO and Gsk-3 inhibition.

Nevertheless, and in line with our findings for decreased ROS and lipofuscin cellular levels after long-term cell exposure to 6BIO, we observed a significant reduction (as compared to controls) in genotoxic stress-related γH_2_A.X foci in the nuclei of cells treated for a long-term period with 6BIO (Fig. 6E). Taken together, these findings suggest that in spite of the reduced rate of cell cycling and the pleiotropic effects of 6BIO in normal human fibroblasts transcriptome; 6BIO treated cells likely senesce in the absence of main hallmarks of cellular senescence, namely increased oxidative stress and accumulation of biomolecular damage.

## Discussion

Cellular senescence is defined as an irreversible growth arrest of the cell cycle and is characterised by (among others) the accumulation of damaged/dysfunctional biomolecules^37–40^. Loss of proteome homeodynamics (proteostasis) is considered to be a hallmark of both cellular senescence and *in vivo* ageing and is marked by the appearance of non-native proteins or protein aggregates in various cell types and tissues^2,3^. Considering that genetic interventions or caloric restriction cannot be applied to humans, there is a strong interest nowadays in identifying dietary NPs (e.g. extracts or isolated compounds) which activate cell protective mechanisms modulating thus healthspan and/or lifespan^1,41^.

We report herein that treatment of skin fibroblasts with the hemi-synthetic compound 6BIO reduced the intracellular ROS levels and conferred significant protection against oxidative stress-mediated genotoxicity. It is well established that cellular senescence and *in vivo* ageing are characterised by increased oxidative stress, decline of proteasome expression and activity, as well as by high rates of genome/proteome instability^3,10,16,20^. It has been thus proposed that activation of PN modules could confer healthspan and/or lifespan extension^4,41,42^. Indeed, higher activity of the PN modules seems to delay the age-related accumulation of stressors in cells resulting in increased healthspan^19^. This assumption is supported by findings indicating that forced reinvestment of resources from the germ line to the soma of *C*. *elegans* promotes higher somatic proteasome activities, clearance of damaged proteins and increased longevity^43^, while, as we and others showed, *Drosophila* reproductive tissues age at significantly lower rates (as compared to the soma) since they exhibit higher capacity to prevent accumulation of damaged proteins due to increased intrinsic proteasome activities^16,44^. 6BIO is a promising chemical scaffold towards this end, since, apart from suppressing DNA damage, it was also found to activate all major PN modules, namely UPS, ALP and molecular chaperones genes; 6BIO also induced the expression of proteasome protein subunits and activated proteasome peptidase activities.

Meijer and colleagues have demonstrated that 6BIO is a highly specific Gsk-3 inhibitor^25^. Gsk-3 is a pleiotropic serine/threonine kinase originally identified as a negative regulator of Glycogen synthase, the rate limiting enzyme in glycogen synthesis^45,46^. Reportedly, Gsk-3 has been also implicated in many other critical cellular functions including cell cycle control, proliferation, differentiation and apoptosis^26–28^. It is not thus surprising that aberrant Gsk-3 expression and/or activity has been involved in many age-related diseases including cancer pathology, as well as neurological, metabolic and immunological disorders^28,30–32^. Our data showed that the 6BIO-mediated inhibitory effect on Gsk-3 kinase activity triggered a feedback regulatory loop aiming to restore physiological Gsk-3 kinase activity levels; this loop resulted in *gsk-3β* gene and protein upregulation and it also reduced the levels of the inhibitory Gsk-3 Ser^21/9^ phosphorylation, likely by suppressing the Akt inhibitory effect on Gsk-3^27^. In line with this finding, it was previously reported that inhibition of Gsk-3 through 6BIO in SH-SY5Y cells led to increased Gsk-3β expression levels^25^. This finding is particularly significant for the drug development research pipeline, since it clearly indicates that human cells are equipped with numerous sensors and responses to counteract disturbances and return to an evolutionary preset species-specific “*ideal*” equilibrium status.

Apart from activating PN modules 6BIO was also found to enhance Nrf2 nuclear localisation; this effect correlated with upregulation of main transcriptional Nrf2 targets including proteasomal genes^14,17^, as well as genes involved in cellular antioxidant responses (i.e. *nqo1*, *txnrd1* ^12^;). In line with this finding we noted a significant decrease in cellular ROS levels. Our findings coincide with previous reports showing that apart from redox interactions with Keap1^12^, Nrf2 is also regulated by Gsk-3. More specifically, Gsk-3 can target Nrf2 directly for β-TrCP-mediated proteasomal degradation^36^ or indirectly by phosphorylating Fyn at threonine residue(s), which then accumulates in the nucleus and phosphorylates Nrf2 at tyrosine 568. This event then results in nuclear export, ubiquitination and degradation of Nrf2^35^. Thus, it is assumed that effective 6BIO-mediated Gsk-3 inhibition results in stabilisation, nuclear localisation and transcriptional activation of Nrf2 downstream gene targets. Likely, the herein reported 6BIO-mediated effect on chaperones (e.g. *hsp70* gene) upregulation correlates to the reported Gsk-3 inhibitory action on the transcriptional activity of heat shock factor-1^47,48^.

The role of either Gsk-3β or 6BIO in the rate of cellular senescence evolvement remained largely obscure. The herein presented data in normal human cells are in agreement with our recent findings showing that administration of 6BIO in *Drosophila* flies activated proteostatic modules, reprogrammed cellular bioenergetics pathways and exerted *in vivo* anti-ageing effects^49^. In support, lithium (a drug acting as Gsk-3 inhibitor) was found to increase flies’ lifespan and resistance to xenobiotics^21^ and to also extend *Caenorhabditis elegans* longevity^50^. Notably however, beyond the beneficial effects of 6BIO in PN and cellular antioxidant responses, we also noted that long-term cell treatment with 6BIO (at non toxic concentration) reduced the rate of cell cycling. This finding could be attributed to the additional reported role of 6BIO as a CDKs inhibitor^34^; furthermore, Ser^114^ phosphorylation of p21 protein by Gsk-3β is required for its degradation in response to UV irradiation, suggesting that Gsk-3β, likely, directly regulates p21 stability^51^. Worth mentioning is also that complete absence of Gsk-3 is toxic in various model organisms^52–54^, while homozygous knockout of Gsk-3β in mice results in embryonic lethality^52,55^. Thus, in accordance to our findings only moderate Gsk-3 inhibition would be beneficial in the long-term.

By studying the short- and long-term effects of 6BIO in human cells transcriptome we noted that following short-term treatment, 6BIO promoted the upregulation of genes involved in DNA damage responses and cell survival regulation, as well as in proteostasis regulation (e.g. molecular chaperones and genes of UPS and ALP). It also induced (among others) genes involved in antioxidant responses, as well as in mitochondrial, metabolic, hypoxia and extracellular matrix-related pathways; these findings clearly demonstrate the pleiotropic effects of Gsk-3 inhibition. In relation to 6BIO-mediated effects in metabolic pathways it was shown previously that 6BIO may also inhibit 3-phosphoinositide dependent protein kinase-1 (Pdpk1)^56^, a major effector of the InS/IGF-1 pathway^57^. Furthermore, we recently reported that 6BIO modulated cellular bioenergetic and metabolic pathways; and it decreased lipid and glucose load in *Drosophila* flies’ tissues^49^. In support, lithium improved mitochondrial energetics in *C*. *elegans* ^58^ and it was shown that Nrf2 positively regulates mitochondria functionality and dynamics^59^ (our unpublished data). In addition to these effects on cellular energetics and metabolism, Gsk-3 also acts as a main negative regulator of glucose homeodynamics through glycogen synthesis inhibition^27^. Thus, the shown effects of 6BIO on bioenergetic and metabolic pathways are not surprising; nevertheless, the mechanistic details of these observations remain to be elucidated. Notably, after long-term cell exposure to 6BIO we observed that in most cases the induction of the assayed genes was less intense (compared to short-term treatment) or was even tend to be inverted. The latter effect referred to (among others) the pro-apoptotic *puma*, *noxa* and *bax* genes, the *hsp27* and *hspa1b* chaperone genes, as well as to the antioxidant *nqo1* and *txnrd1* genes, and to metabolic *pdpk1*, *akt1* and *pdk1* genes. The trend for pro-apoptotic genes downregulation which occurs while the *bcl2* gene remains upregulated further support the long-term cytoprotective role of 6BIO in normal human cells.

Interestingly enough, we also found that long-term treatment of normal human cells with 6BIO significantly suppressed (as compared to control cells) the rate of senescence-associated accumulation of biomolecular damage; a well established hallmark of cellular senescence^1,2,38^ and an emerging hallmark of tumorigenesis^60^. This finding indicates that 6BIO could also exert anti-tumor activities. Indeed, two other herein reported observations support a potential anti-tumor activity of 6BIO. Firstly, we noted that long-term incubation of fibroblasts with 6BIO altered cell morphology. Specifically, the mesenchymal-like shaped fibroblasts tend to become more epithelial-like in shape indicating a process of mesenchymal-epithelial transition (MET). In line with this observation, studies of Kroon and colleagues also revealed MET in PC-3M-Pro4 cells after inhibition of Gsk-3^61^. Secondly, and in agreement with previous similar findings^62,63^, we noted that 6BIO-mediated Gsk-3 inhibition suppressed fibroblasts rate of migration; thus, 6BIO could also exert anti-metastatic activities.

Taken together, our reported findings (Suppl. Fig. S9) suggest that 6BIO (and likely other indirubin molecules from edible gastropod mollusks and/or plants sources) activates cytoprotective modules. Thus, it has the potential to enter the challenging field of translational medicine, as its scaffold can be used for the development of novel anti-ageing (and likely anti-tumor) compounds.

## Materials and Methods

### Chemicals and 6BIO synthesis

All chemicals were purchased from Sigma. Microwave-assisted reactions were performed in a single mode CEM apparatus; High Resolution Mass Spectrometry (HRMS) Spectra were determined on a MSQ Orbitrap Thermofinnigan spectrometer and Nuclear magnetic resonance (NMR) spectra on a Brucker Avance 600 spectrometer (600 MHz). 6BIO was synthesised as previously described^34^ and analytical data (HRMS and NMR) were conformed to literature. Purification of the synthesised product was conducted using flash silica gel 60 (40–63 μm) from Merck; the purity of the synthesised compound has been determined by High-performance liquid chromatography (HPLC) and was above 95%.

### Cell lines and cell culture conditions

Human newborn foreskin (BJ cells) and human lung embryonic (IMR90 cells) fibroblasts were purchased from the American Tissue Culture Collection. Cells were cultured in Dulbecco’s modified Eagle’s medium (ThermoFisher Scientific), supplemented with 10% (v/v) fetal bovine serum, 2 mM glutamine and 1% non-essential amino acids, in a humidified incubator at 5% CO_2_ and 37 °C. In all experimental procedures applied proliferating cells were subcultured at a split ratio 1:2 (when confluent) by using a trypsin/EDTA solution (ThermoFisher Scientific). For the long-term experiments, 6BIO was used at the non-cytotoxic concentration of 1 μM; cells were cultured with 6BIO for 100–150 days and the culture medium was renewed every two days. Cell treatment with the various agents used was done as described in Figure legends; H_2_O_2_ was purchased from AppliChem and DXR from Sigma.

### Cell survival assay

The effect of 6BIO on the viability of BJ cells was examined using the MTT assay. Briefly, cells were plated in flat-bottomed 96-well microplates and the next day they were incubated with different concentrations of 6BIO for 24 h. Afterwards, the medium was replaced by 3-(4,5-dimethylthiazol-2-yl)-2,5-diphenyltetrazolium bromide (MTT, Sigma-Aldrich) dissolved at a final concentration of 1 mg/ml in serum-free, phenol red-free medium. The formed formazan crystals were then dissolved by isopropanol and the absorbance of the solution was measured at 570 nm wavelength. Survival of control cells was arbitrarily set to 100%.

### RNA Isolation and Quantitative Real Time PCR (Q-RT-PCR) analysis

Total RNA was isolated using the TRI Reagent® RNA Isolation Reagent (Sigma-Aldrich) and quantified with BioSpec-nano spectrophotometer (Shimadzu Inc.). Subsequently, cDNA synthesis and Q-RT-PCR were performed using the Maxima First Strand cDNA Synthesis Kit for RT-qPCR (ThermoFisher Scientific) and the Maxima SYBR Green/ROX qPCR Master Mix (ThermoFisher Scientific), respectively. Primers were designed using the primer-BLAST tool (http://www.ncbi.nlm.nih.gov/tools/primer-blast/) and are described in Supplemental Information.

### Immunofluorescence antigen staining and detection of lipofuscin autofluorescence

Cells grown on coverslips were fixed with 4% formaldehyde in PBS and permeabilised with 0.2% Triton X-100. After blocking (3% BSA in PBS), cells were sequentially incubated with primary and secondary antibodies for 1 hr at room temperature. For visualising nuclei and F-actin cells were counterstained with DAPI and Alexa Fluor Phalloidin (ThermoFisher Scientific), respectively. Samples were viewed at a NIKON C1 Confocal Laser Scanning Microscope (CLSM). The total pan-nuclear phospho-H_2_A.X^Ser139^ (γH_2_A.X) signal per cell in captured images was quantified by Image J software.

Lipofuscin autofluorescence of control (DMSO) and 6BIO treated cells (IMR90 and BJ cells) was detected by viewing fixed cells at an Eclipse TS-100F NIKON inverted fluorescent microscope equipped with NIKON DS camera.

### Measurement of Reactive Oxygen Species (ROS)

For the assessment of ROS production, cells were incubated with 10 μM CM-H_2_DCFDA dye (ThermoFisher Scientific) in PBS for 30 min at 37 °C in the dark. Following dye removal, cells were incubated for 10 min with PBS and then, either were stained with DAPI and viewed at the NIKON C1 CLSM or the produced fluorescence was measured using the Infinite 200 Tecan microtiter-plate photometer (Tecan Trading AG, Switzerland) at excitation and emission wavelengths of 490 and 520 nm, respectively.

### Measurement of proteasome peptidase activities

Proteasome peptidase activities in human skin fibroblasts were measured at a VersaFluor^TM^ Fluorometer System (Bio-Rad laboratories, Hercules, CA, USA) as described previously^64^ by using the Boc-Leu-Arg-Arg-AMC-LRR (trypsin-like activity, T-L), Z-Leu-Leu-Glu-AMC-LLE (caspase-like activity, C-L) and Suc-Leu-Leu-Val-Tyr-AMC-LLVY (chymotrypsin-like activity, CT-L) (Enzo Life Sciences, Inc. Farmingdale, NY, USA) fluoropeptides.

### Preparation of cell extracts and immunoblotting analysis

Cells were lysed on ice in NP-40 lysis buffer (150 mM NaCl, 1% NP-40, 50 mM Tris pH 8.0) containing protease and phosphatase inhibitors and lysates were cleared by centrifugation for 10 min at 19 000 × g (4 °C). The total protein content of each lysate was measured by Bradford assay (Bio-Rad).

Immunoblotting was done as described previously^64^. Briefly, equal total protein μg per sample were separated by SDS-PAGE and blotted onto a nitrocellulose membrane (Immobilion-P, Millipore, Eschborn, Germany); primary and horseradish peroxidase-conjugated secondary antibodies were applied for 1 hr at room temperature and immunoblots were developed by an enhanced chemiluminescence reagent kit (Santa Cruz Biotechnology).

### Cell migration assay

The migration capacity of treated (or control) cells was evaluated by using the *in vitro* scratch assay, as described previously^65^. Briefly, cells were seeded onto 60 mm dishes and when confluent they were scratched by a 200 µl sterile pipette tip. After removing detached cells by washing once with culture medium, remaining cells were incubated with fresh medium containing (or not) 6BIO until cells from an experimental condition closed the gap. Cell cultures were recorded at 24 and 48 h by using phase contrast optics at an Eclipse TS-100F NIKON inverted microscope equipped with NIKON DS camera.

### siRNA transfection experiments

Cells were transfected for 24 to 48 h using DharmaFECT Transfection reagent, as well as the SMARTpool ON-TARGET plus Gsk-3β siRNA (L-003010-00-0005) or the ON-TARGET plus non-targeting pool (siCtrl) (D-001810-10-05) (GE Healthcare Dharmacon Inc.) according to manufacturer’s instructions.

### Antibodies used

Primary antibodies against Nrf2 (sc-722), proteasome α7 subunit (sc-100456), proteasome β5 subunit (sc-55009), Beclin1 (sc-11427), Gsk-3β (sc-9166), p53 (sc-47698), p21^WAF1/CIP1^ (sc-6246), Actin (sc-1615) and GAPDH (sc-25778), as well as the horseradish peroxidase-conjugated secondary antibodies were purchased from Santa Cruz Biotechnology. The antibodies against phospho-Histone H_2_A.X^Ser139^ (9718), phospho-Gsk-3α/β^Ser21/9^ (9331) and phospho-p53^Ser15^ (9286) were obtained from Cell Signaling Technology. The antibodies against proteasome Rpn6/PSMD11 (NBP1-46191) subunit, Rpt6 (BML-PW9265) subunit and p16^INK4a^ (550834) were purchased from Novus Biologicals, Enzo Biochem Inc. and BD Biosciences, respectively. The secondary FITC-conjugated IgG antibody (711-095-152) was purchased from Jackson ImmunoResearch Laboratories, Inc.

### Statistical analysis

Experiments were performed at least in duplicates. For statistical analyses MS Excel was used. Statistical significance was evaluated using one-way analysis of variance (ANOVA). Data points correspond to the mean of the independent experiments and error bars denote standard deviation (SD); significance at P < 0.05 or P < 0.01 is indicated in graphs by one or two asterisks, respectively.

### Data availability statement

The datasets generated during and/or analysed during the current study are available from the corresponding author on reasonable request.

## Electronic supplementary material

Supplementary Information
