# Supplementary material for: 6-bromo-indirubin-3′-oxime (6BIO), a Glycogen synthase kinase-3β inhibitor, activates cytoprotective cellular modules and suppresses cellular senescence-mediated biomolecular damage in human fibroblasts
Source: Sci Rep. 2017 Sep 15;7:11713. doi: 10.1038/s41598-017-11662-7 (PMC5601901; doi:10.1038/s41598-017-11662-7)
Supplement: Supplementary file 1 — Supplementary Information [file 41598_2017_11662_MOESM1_ESM.pdf]

6-bromo-indirubin-3′-oxime (6BIO), a Glycogen synthase kinase-3β inhibitor, activates cytoprotective cellular modules and suppresses cellular senescence-mediated biomolecular damage in human fibroblasts

Aimilia D. Sklirou, Nicolas Gaboriaud-Kolar, Issidora Papassideri, Alexios-Leandros Skaltsounis and Ioannis P. Trougakos

Supplemental Information

Experimental procedures

Quantitative Real Time PCR (Q-RT-PCR) primers

The primers used for Q-PCR were as follows (F: forward, R: reverse, Sequence: 5'→3').
H-*atm*-F: TGA-GAA-ACT-CTC-AGG-AAA-CTC-TGT-T, H-*atm*-R: TCA-GAG-TAG-GGT-GAA-GCT-CAG-T; H-*tp53*-F: CGC-TTC-GAG-ATG-TTC-CGA-GA, H-*tp53*-R: CGG-GAG-GTA-GAC-TGA-CCC-TT; H-*p21*-F: GAC-CAT-GTG-GAC-CTG-TCA-CT, H-*p21*-R: CTT-CCT-GTG-GGC-GGA-TTA-GG; H-*foxo3*-F: CGA-GAG-CTC-CCC-GGA-CAA, H-*foxo3*-R: TTC-TGG-ACC-CGC-ATG-AAT-CG; H-*puma*-F: GAA-GAG-CAA-ATG-AGC-CAA-ACG, H-*puma*-R: GGA-GCA-ACC-GGC-AAA-CG; H-*noxa*-F: ACT-GTT-CGT-GTT-CAG-CTC, H-*noxa*-R: GTA-GCA-CAC-TCG-ACTT-CC; H-*bax*-F: GAG-AGG-TCT-TTT-TCC-GAG-TGG, H-*bax*-R: CCT-TGA-GCA-CCA-GTT-TGC-TG; H-*bcl2*-F: GGA-TCC-AGG-ATA-ACG-GAG-GC, H-*bcl2*-R: GAA-ATC-AAA-CAG-AGG-CCG-CA; H-*grp78*-F: AGG-AAC-CAT-CCC-GTG-GCA-TA, H-*grp78*-R: TGA-CAC-CTC-CCA-CAG-TTT-CA; H-*chop*-F: CGA-CAG-AGC-CAA-AAT-CAG-AGC, H-*chop*-R: TTC-AGG-TGT-GGT-GAT-GTA-TGA-AGA; H-*ubl5*-F: TTT-TAA-GGA-CCA-CGT-GTC-TCT-GGG, H-*ubl5*-R: GGG-GCA-GGA-AGA-TGA-GGA-TTC; H-*hspa1a/hsp70-1*-F: TAA-CCC-CAT-CAT-CAG-CGG-AC, H-*hspa1a/hsp70-1*-R: AGC-AAT-CTT-GGA-AAG-GCC-CC; H-*hspa9*-F: CGA-CAC-AGA-AAC-CAA-GAT-GGA-AG, H-*hspa9*-R: CGC-TGT-CTT-TTC-TAG-CCA-GG; H-*stub1*-F: TAC-GGC-CGC-GCG-ATC-A, H-*stub1*-R: GAA-GTG-CGC-CTT-CAC-AGA-CT; H-*psma3*-F: GCT-GGG-TTG-GTG-AAT-TAA-CTA-ATG-G, H-*psma3*-R: GGG-CTA-AAT-AGT-TAC-ATT-GGA-CTG-G; H-*psmb6*-F: GAC-CTG-ATG-GCG-GGA-ATC-AT, H-*psmb6*-R: CAA-AGC-GAG-AGC-ATT-GGC-A; H-*psmb7*-F: CAA-GCT-GGA-TTT-TCT-CCG-CC, H-*psmb7*-R: TGA-TTT-TCT-CAG-TGA-GGA-CTG-C; H-*atg5*-F: GGC-CAT-CAA-TCG-GAA-ACT-CA, H-*atg5*-R: CCT-AGT-GTG-TGC-AAC-TGT-CCA; H-*atg7*-F: CCG-TCA-GCC-TGG-CAT-TTG-AC, H-*atg7*-R: TCA-GCA-GCT-TGG-GTT-TCT-TGA-T; H-*becn1*-F: AAC-CAG-ATG-CGT-TAT-GCC-CA, H-*becn1*-R: TCC-ATT-CCA-CGG-GAA-CAC-TG; H-*maf*-F: AAA-TAC-GAG-AAG-CTG-GTG-AGC-

AA, H-*maf*-R: CGG-GAG-AGG-AAG-GGT-TGT-C; H-*atp5b*-F: TTG-CTG-AGG-TCT-TCA-CAG-GT, H-*atp5b*-R: CCA-CAG-CTT-CTT-CAA-TGG-GTC; H-*atp5h*-F: CCA-CTT-CCG-TTA-CTT-GCT-GC, H-*atp5h*-R: CAA-GTT-TTC-GCC-CAG-CCA-TT; H-*sdha*-F: CGC-TGC-AGA-CCA-TCT-ACG-G, H-*sdha*-R: GTG-TGC-TTC-CTC-CAG-TGC-TC; H-*timm17a*-F: CCA-CTT-CCG-TTA-CTT-GCT-GC, H-*timm17a*-R: CAA-GTT-TTC-GCC-CAG-CCA-TT; H-*timm17b*-F: TGA-GAG-GTA-GTG-CCA-ATG-CTG, H-*timm17b*-R: ACA-GTC-GAT-GGT-GGA-GAA-CAG; H-*tfam*-F: AGA-TTC-CAA-GAA-GCT-AAG-GGT-GA, H-*tfam*-R: CAG-AGT-CAG-ACA-GAT-TTT-TCC-AGT-T; H-*pprc1*-F: AGT-GGT-TGG-GGA-AGT-CGA-AG, H-*pprc1*-R: CCT-GCC-GAG-AGA-GAC-TGA-C; H-*ppargc1a*-F: CTT-TGC-GCA-GGT-CAA-ACG-AA, H-*ppargc1a*-R: GGT-GGA-AGC-AGG-GTC-AAA-GT; H-*ppargc1b*-F: TGC-TGG-CCC-AGA-TAC-ACT-GA, H-*ppargc1b*-R: TTC-CTC-GAG-GGT-TAA-GGC-TG; H-*insr*-F: CAG-CCT-TAC-CAA-GGC-CTG-TC, H-*insr*-R: AAG-GTT-GGC-CTC-ATC-TTG-GG; H-*pdpk1*-F: CCG-TTC-TTC-GAG-TCC-GTC-AC, H-*pdpk1*-R: AGC-CAA-ACT-GGC-TCA-GGA-GA; H-*akt1*-F: TCT-TTG-CCG-GTA-TCG-TGT-GG, H-*akt1*-R: GTG-ATC-ATC-TGG-GCC-GTG-AA; H-*mtor*-F: GTG-AAA-CCA-GAG-GCC-CTA-AAT-A, H-*mtor*-R: TAG-CAC-TGG-CAG-AGG-TTT-TCA-T; H-*gsk3a*-F: GGC-TCA-TTT-GGG-GTC-GTG-TA, H-*gsk3a*-R: CAG-TGG-TCC-AGC-TTA-CGC-AT; H-*gsk3b*-F: ACT-GTA-ACA-TAG-TCC-GAT-TGC-GT, H-*gsk3b*-R: GCG-TCT-GTT-TGG-CTC-GAC-TA; H-*gys1*-F: TGATTCCTGCTCGCAGCTCA, H-*gys1*-R: CCTCGTTGGGCTCGTAGGTG; H-*foxo1*-F: AGT-GGA-TGG-TCA-AGA-GCG-TG, H-*foxo1*-R: CTT-GCT-GTG-TAG-GGA-CAG-ATT; H-*pepck*-F: GAA-GTG-CTT-TGC-CCT-ACG-C, H-*pepck*-R: CCC-CCA-CAC-ACT-CCA-CTT-TC; H-*pdk1*-F: CCC-TAG-AGG-GTT-ACG-GGA-CA, H-*pdk1*-R: GCC-TCG-TGG-TTG-GTG-TTG-TA; H-*pkm*-F: TAG-TTC-TGA-CGG-AGT-CTG-GC, H-*pkm*-R: GAC-GAG-CTG-TCT-GGG-GAT-TC; H-*vegfa*-F: CGAGGCAGCTTGAGTTAAACG, H-*vegfa*-R: GGTGAGAGATCTGGTTCCCG; H-*hif1a*-F: GCCAGACGATCATGCAGCTA, H-*hif1a*-R: ATCCATTGATTGCCCCAGCA; H-*mmp2*-F: ATA-ACC-TGG-ATG-CCG-TCG-TG, H-*mmp2*-R: AGC-CTA-GCC-AGT-CGG-ATT-TG; H-*il8*-F: ACC-ACC-GGA-AGG-AAC-CAT-CT, H-*il8*-R: GCA-CTG-ACA-TCT-AAG-TTC-TTT-AGC-A. The primers used for *psma7*, *psmb1*, *psmb2, psmb5*, *rpn6*, *rpn11*, *sqstm1*, *hdac6*, *ctsl*, *ctsd*, *hsf1*, *hsp27*, *hspa1b/hsp70-2, hsp90*, *clu*, *nrf2*, *keap1*, *nqo1*, *txnrd1* genes were as described in Sklirou et al. (2015). The beta-2-microglobulin gene (H-*b2m*-F: ACT-GAA-TTC-ACC-CCC-ACT-GA, H-*b2m*-R: AAG-CAA-GCA-AGC-AGA-ATT-TGG) was used as Q-PCR normaliser.

Full names of analyzed genes

atm: ataxia telangiectasia mutated, *tp53*: tumor protein p53, *p21*: cyclin-dependent kinase inhibitor 1A, *foxo3*: forkhead box O3, *puma*: BCL2 binding component 3, *noxa*: phorbol-12-myristate-13-acetate-induced protein 1, *bax*: BCL2-associated X protein, *bcl2*: B-cell CLL/Lymphoma 2, *grp78/ hspa5*: heat shock protein family A (Hsp70) member 5, *chop/ddit3*: DNA-damage inducible transcript 3, *ubl5*: ubiquitin-like 5, *hsf1*: heat shock transcription factor 1, *hsp27/hspb1*: heat shock protein

family B (small) member 1, *hspa1a/hsp70-1*: heat shock protein family A (Hsp70) member 1A, *hspa1b/hsp70-2*: heat shock protein family A (Hsp70) member 1B, *hsp90/hsp90aa1*: heat shock protein 90 alpha family class A member 1, *clu/apoj*: clusterin/ apolipoprotein J, *stub1*: STIP1 homology and U-Box containing protein 1, *hspa9*: heat shock protein family A (Hsp70) member 9, *psma3*: proteasome subunit alpha type 3, *psma7*: proteasome subunit alpha type 7, *psmb1*: proteasome subunit beta type 1, *psmb2*: proteasome subunit beta type 2, *psmb5*: proteasome subunit beta type 5, *psmb6*: proteasome subunit beta type 6, *psmb7*: proteasome subunit beta type 7, *rpn6/ psmd11*: proteasome 26S subunit, non-ATPase, 11, *rpn11/psmd14*: proteasome 26S subunit, non-ATPase, 14, *atg5*: autophagy related 5, *atg7*: autophagy related 7, *becn1/atg6*: beclin 1, autophagy related 6, *sqstm1/p62*: sequestosome 1, *hdac6*: histone deacetylase 6, *ctsl:* cathepsin L, *ctsd*: cathepsin D, *nrf2*: nuclear factor, erythroid 2-like 2, *maf*: musculoaponeurotic fibrosarcoma oncogene, *keap1*: kelch-like ECH-associated protein 1, *nqo1*: NAD(P)H quinone dehydrogenase 1, *txnrd1*: thioredoxin reductase 1, *atp5b*: ATP synthase, H^+ transporting mitochondrial F1 complex, beta polypeptide, *atp5h*: ATP synthase, H^+ transporting, mitochondrial Fo complex subunit D, *sdha*: succinate dehydrogenase complex flavoprotein subunit A, *timm17a*: translocase of inner mitochondrial membrane 17A, *timm17b*: translocase of inner mitochondrial membrane 17B, *tfam*: transcription factor A, mitochondrial, *pprc1*: peroxisome proliferator-activated receptor gamma (PPARG), coactivator-related 1, *ppargc1a*: PPARG coactivator 1 alpha, *ppargc1b*: PPARG coactivator 1 beta, *insr*: insulin receptor, *pdpk1*: 3-phosphoinositide dependent protein kinase 1, *akt1*: AKT serine/threonine kinase 1, *mtor*: mechanistic target of rapamycin, *gsk3a*: glycogen synthase kinase 3 alpha, *gsk3b*: glycogen synthase kinase 3 beta, *gys1*: glycogen synthase 1, *foxo1*: forkhead box O1, *pepc*k: phosphoenolpyruvate carboxykinase 1, cytosolic, *pdk1*: pyruvate dehydrogenase kinase 1, *pkm*: pyruvate kinase, muscle, *vegfa*: vascular endothelial growth factor A, *hif1a*: hypoxia inducible factor 1 alpha subunit, *mmp2*: matrix metallopeptidase 2, *il8*: interleukin 8.

Figures legends

Suppl. Figure S1. At concentrations higher than 5 μM 6BIO shows increased toxicity. Relative (%) survival (MTT assay) of BJ fibroblasts exposed to the indicated concentrations of 6BIO for 24h (values at concentrations up to 5 μM are those shown in Fig. 1). Control samples values were set to 100%. Bars, ± SD; $^*P < 0.05$; $^{**}P < 0.01$.

Suppl. Figure S2. Reduced p53 activation and histone H2A.X phosphorylation [as compared to Doxorubicin (DXR)-treatment] in BJ cells co-treated with both 6BIO and DXR. (A) Expression levels of p53, 12h post-treatment of BJ cells with 1 μM DXR in the presence (or not) of 2 μM 6BIO. (B) Expression levels of p53; the Ser^{15} phosphorylated form of p53 and the Ser^{139} phosphorylated form of histone $H_2A.X$ ($\gamma H_2A.X$) in cells treated for 24h with 1 μM DXR in the presence (or not) of 2 μM

6BIO. Quantitation of analysed proteins' expression levels is shown at the graphs at the right; in control samples values were set to 1. Bars, ± SD. *P < 0.05; **P < 0.01 *vs*. DXR treated cells.

Suppl. Figure S3. Treatment of cells with 6BIO activates a regulatory feedback loop aiming to restore physiological Gsk-3 kinase activity levels. (A) Relative mRNA expression levels (Q-RT-PCR) of the *gsk-3β* gene after exposing cells to the shown concentrations of 6BIO for 24h. (B) Representative blots showing Gsk-3β protein expression levels, as well as the inhibitory Gsk-3$^{Ser21/9}$ phosphorylation levels after treating cells with the shown 6BIO concentrations for 36h. Quantitation of shown blots is presented in Suppl. Fig. S10. The *b2m* gene expression (A) and GAPDH probing (B) were used as reference for RNA and protein input, respectively. Bars, ± SD; **P <0.01 *vs.* controls set to 1.

Suppl. Figure S4. RNAi-mediated Gsk-3β knock down conferred protection against oxidative stress-induced DNA damage. (A_1) Representative CLSM images following immunofluorescence staining of the Ser^{139} phosphorylated form of histone $H_2A.X$ ($\gamma H_2A.X$); cells were treated for 48h with 200 μM H_2O_2 in the presence or absence of control or Gsk-3β siRNAs. (A_2) Relative quantification of the $\gamma H_2A.X$ foci staining intensity per nucleus. Cells nuclei were counterstained with DAPI (blue) and actin with Phalloidin (red). Control samples values were set to 100%. Bars, ± SD; **P < 0.01. Bars in (A_1) 10 μM.

Suppl. Figure S5. Long-term effects of 6BIO on *gsk-3β* gene and protein expression. (A) Q-PCR expression analyses of *gsk-3β* gene after long-term continuous cell incubation with 6BIO. (B) Representative blots showing Gsk-3β protein expression levels after long-term treatment of cells with 6BIO; quantitation of shown blots is presented in Suppl. Fig. S11. The *b2m* gene expression (A) and GAPDH probing (B) were used as reference for RNA and protein input, respectively. Bars, ± SD; *P <0.05 *vs.* controls set to 1.

Suppl. Figure S6. Terminally senescent 6BIO treated cells (prolonged exposure) express lower levels of Nrf2 target genes. Q-PCR expression analyses of the *nrf2* gene and of its transcriptional target genes *keap1*, *nqo1* and *txnrd1* after long-term treatment of BJ cells with 1 μM of 6BIO. *b2m* gene expression was used as reference for RNA input. Bars, ± SD; *P < 0.05; **P < 0.01 *vs.* controls set to 1.

Suppl. Figure S7. Prolonged administration of 6BIO to IMR90 cells significantly reduced the rate of senescence-associated lipofuscin accumulation. Shown images indicate autofluorescence of cellular lipofuscin in IMR90 cells treated (or not) with 6BIO for 30 days. Bars, 50 μM.

Suppl. Figure S8. Q-RT-PCR gene expression analyses after short- or long-term treatment of BJ cells with 1 μM 6BIO. Cells were continuously exposed (or not) to 6BIO for 24h (short-term) or 100 days (long-term). The full names of the assayed 64 genes are reported in Suppl. Experimental Procedures; a relevant heatmap and rectangles indicating minor gene expression alterations within the range of 0.8 fold downregulation up to 1.5 upregulation are shown.

Suppl. Figure S9. Our summarized findings highlight the notion that 6BIO-mediated Gsk-3 inhibition activates cellular antioxidant responses and proteostatic mechanisms. We report that treatment of normal human fibroblasts with the hemi-synthetic compound 6BIO (a Gsk-3 inhibitor) activated antioxidant and proteostatic modules, enhanced resistance to stressors and suppressed senescence-related accumulation of biomolecular damage. Thus, 6BIO represents a promising scaffold for developing novel anti-ageing (and likely anti-cancer) compounds.

Suppl. Figure S10. Quantitative analysis of protein expression levels in the immunoblots indicated (see also the respective Figs). Bars, ± SD; $^{*}P < 0.05$; $^{**}P < 0.01$ *vs.* controls set to 1.

Suppl. Figure S11. Quantitative analysis of protein expression levels in the immunoblots indicated (see also the respective Figs). Bars, ± SD; $^{*}P < 0.05$; $^{**}P < 0.01$ *vs.* controls set to 1.

- Full blots of all the immunoblotting assays presented are shown at the end of the *Supplemental Information* Section.

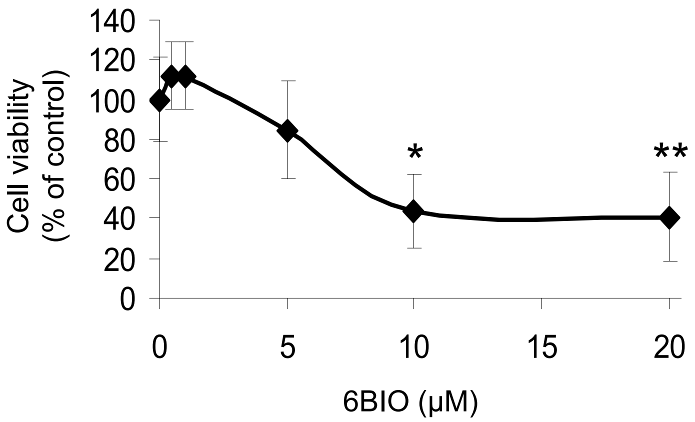

Sklirou et al. Suppl. Fig. S1

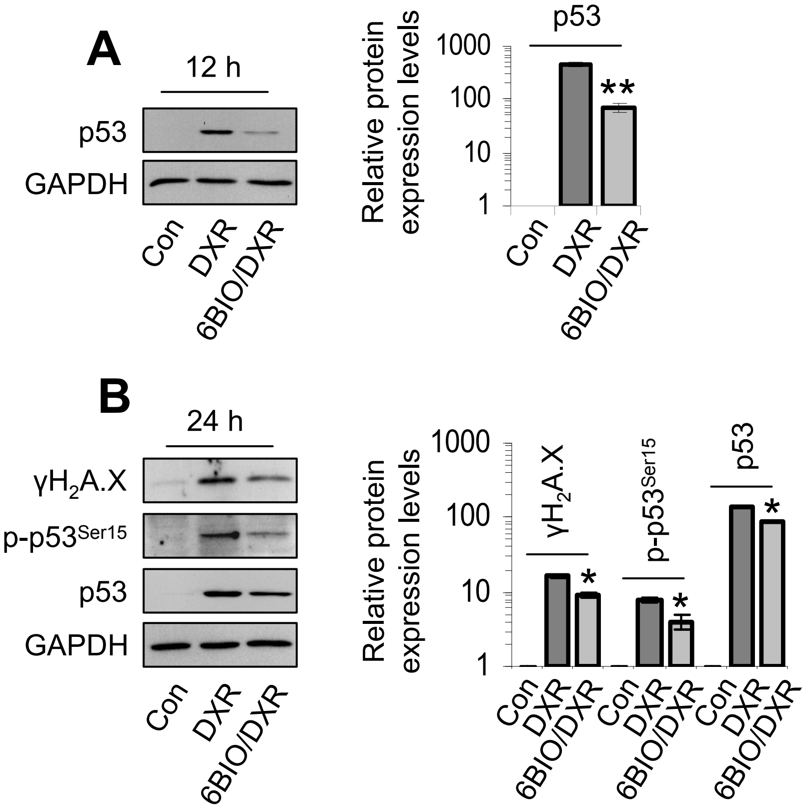

Sklirou et al. Suppl. Fig. S2

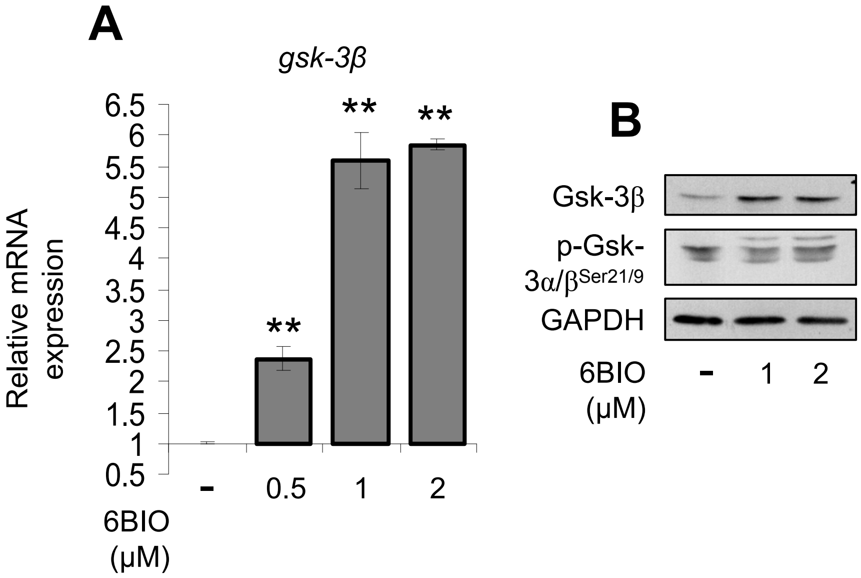

Sklirou et al. Suppl. Fig. S3

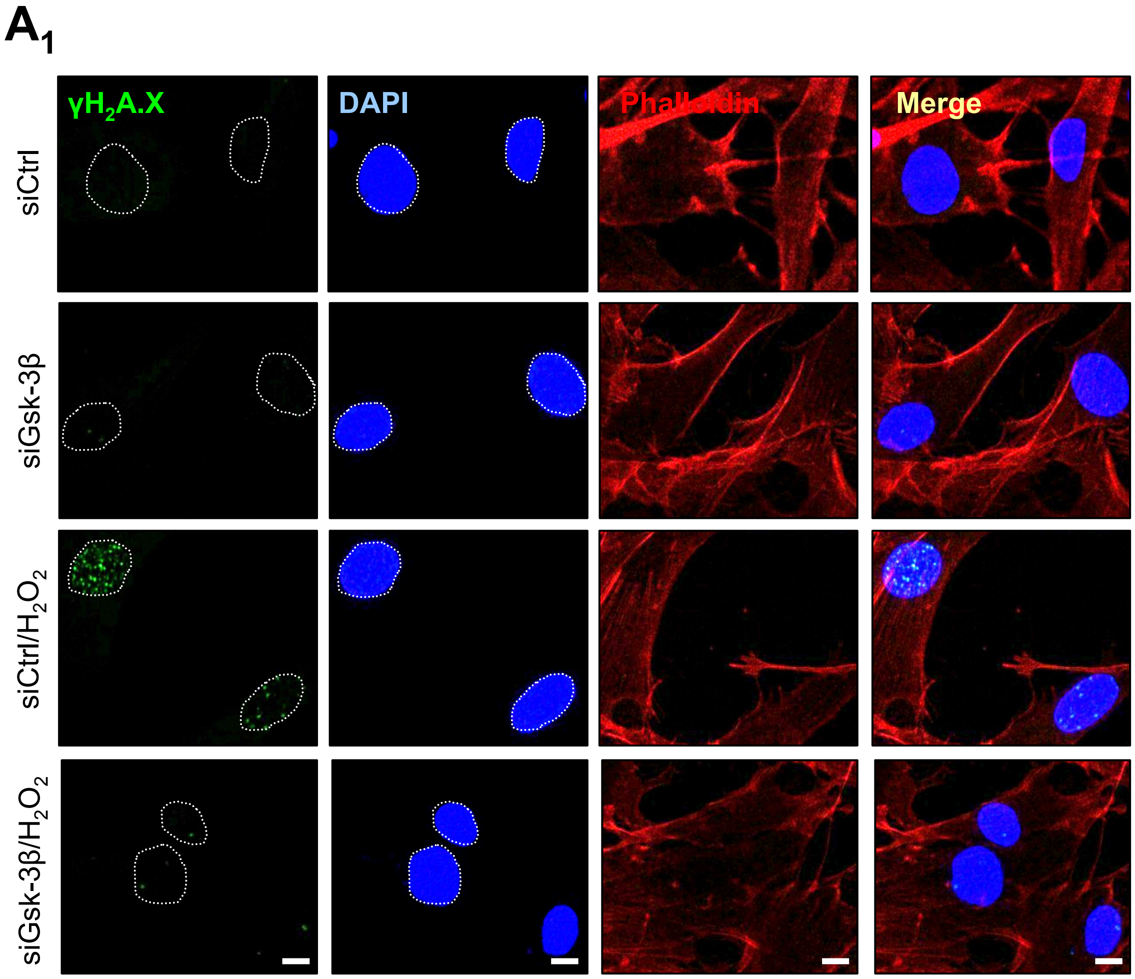

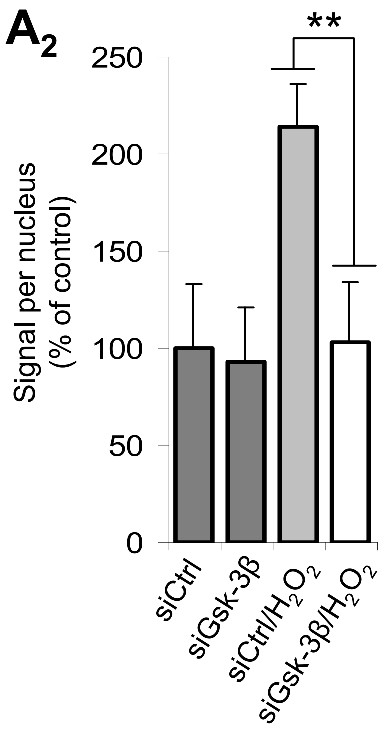

Sklirou et al. Fig. S4

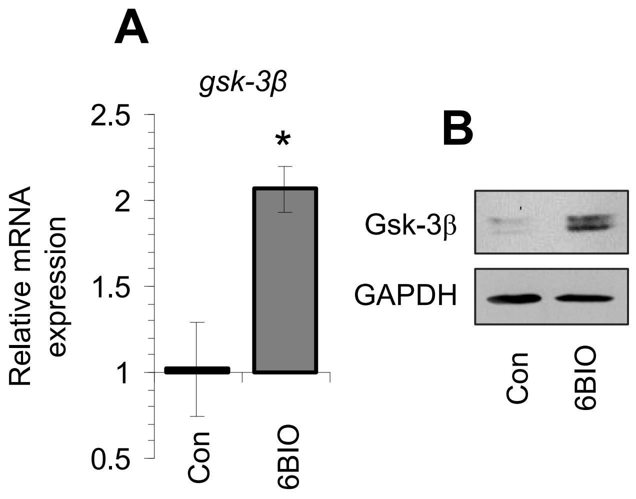

Sklirou et al. Suppl. Fig. S5

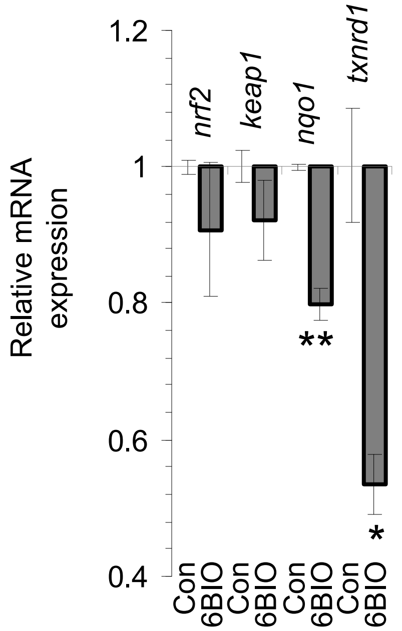

Sklirou et al. Suppl. Fig. S6

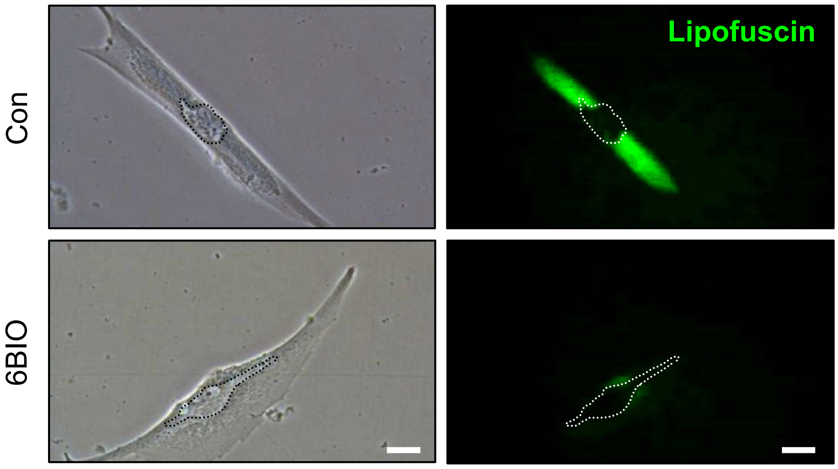

Sklirou et al. Suppl. Fig. S7

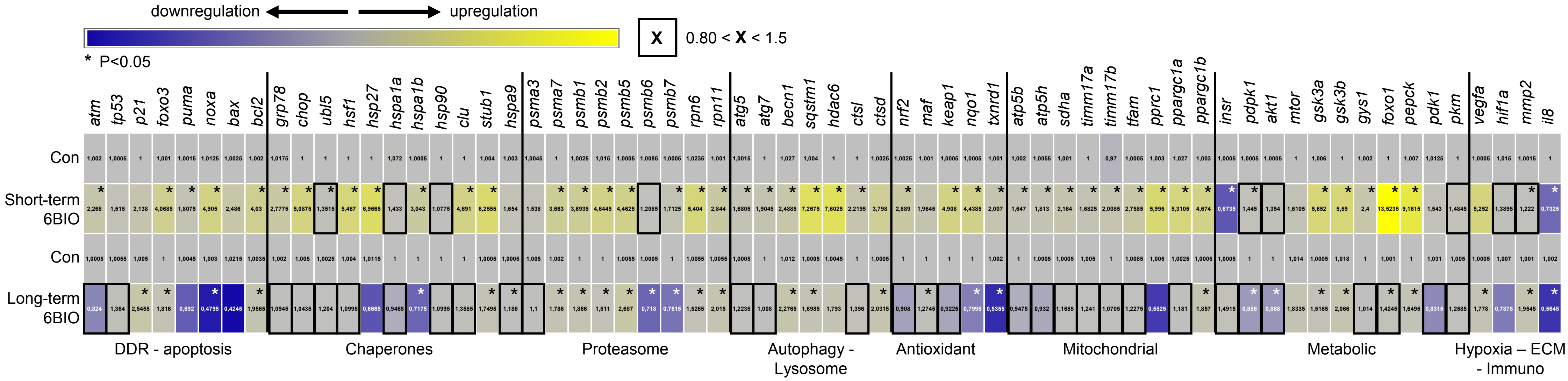

Sklirou et al. Suppl. Fig. S8

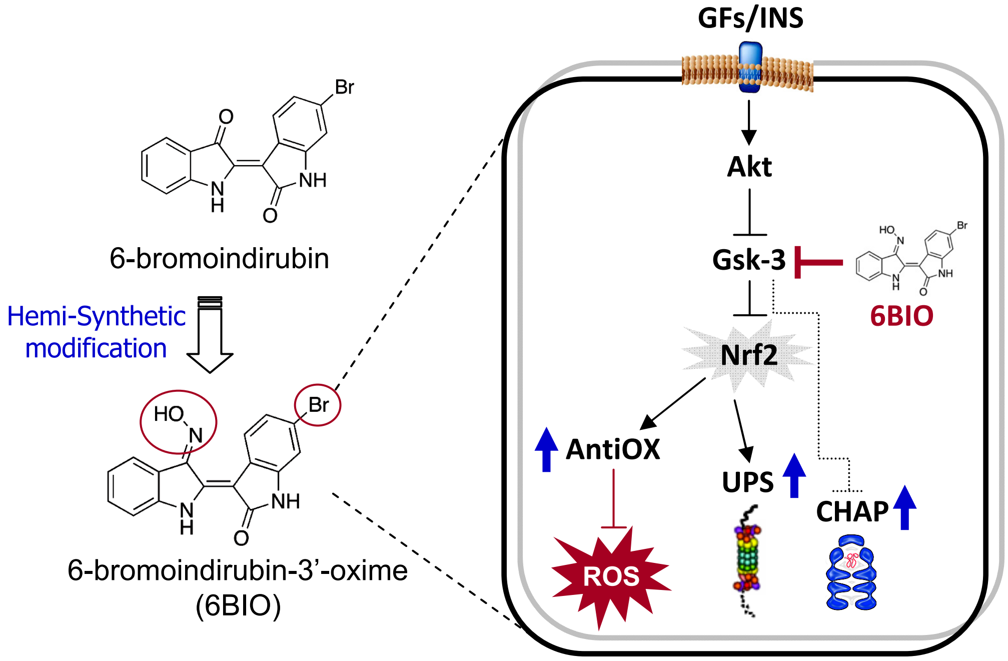

Sklirou et al. Suppl. Fig. S9

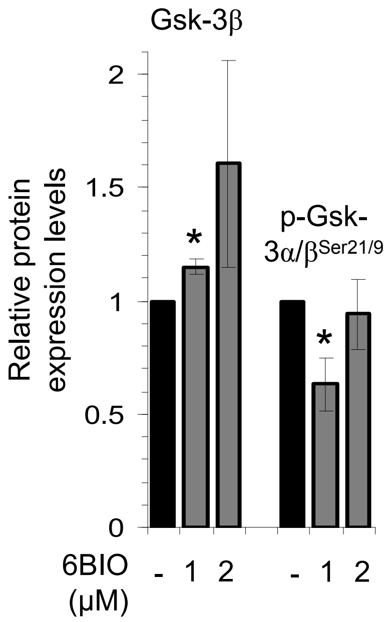

Suppl. Fig. S3B

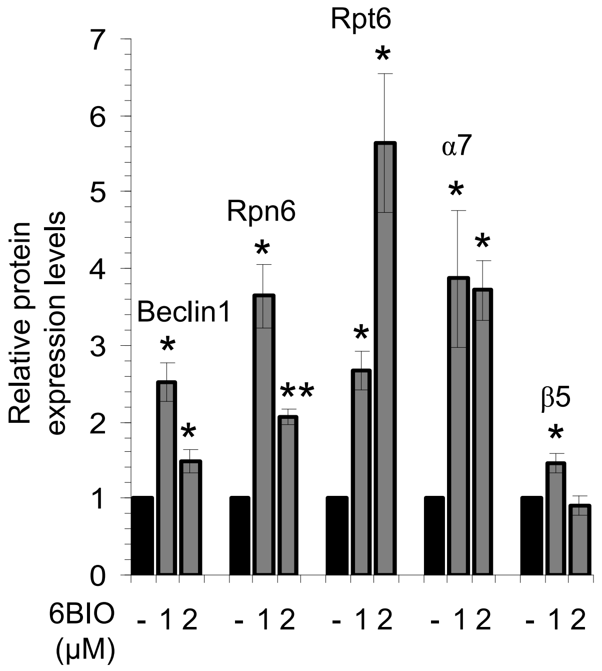

Fig. 2B

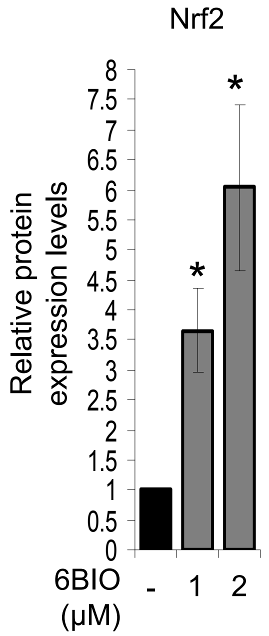

Fig. 3B

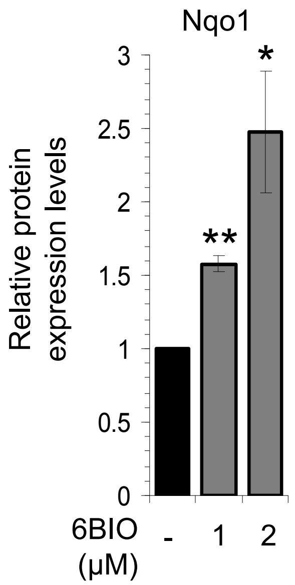

Fig. $3C_2$

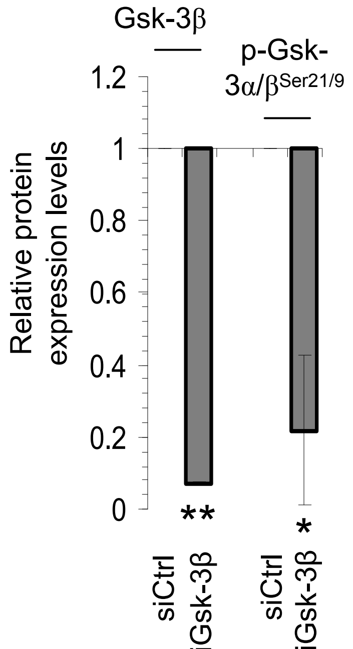

Fig. $4A_2$

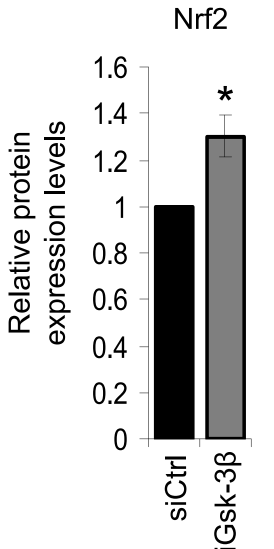

Fig. $4B_2$

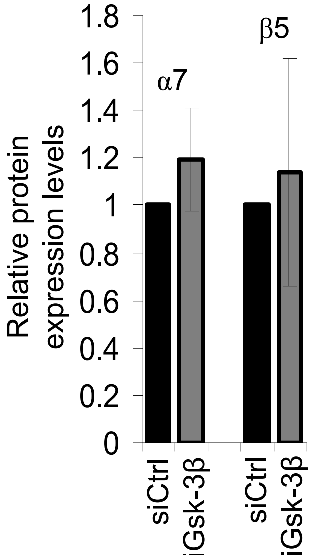

Fig. $4C_2$

Sklirou et al. Suppl. Fig. S10

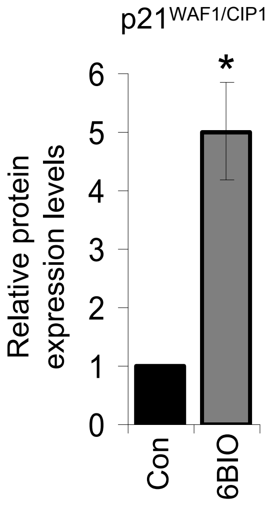

Fig. $5E_2$

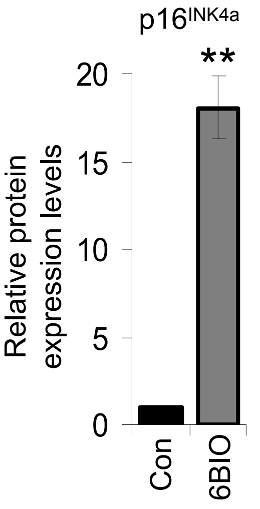

Fig. $5E_2$

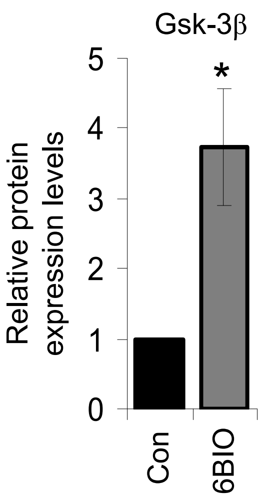

Suppl. Fig. S5B

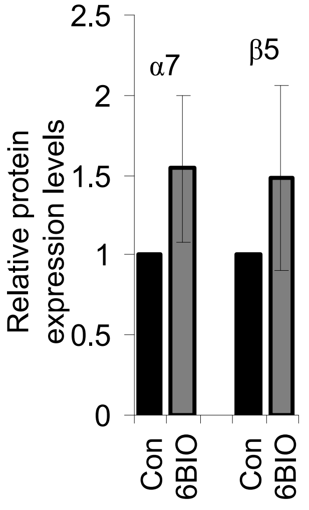

Suppl. Fig. 6A
(upper panel)

Sklirou et al. Suppl. Fig. S11

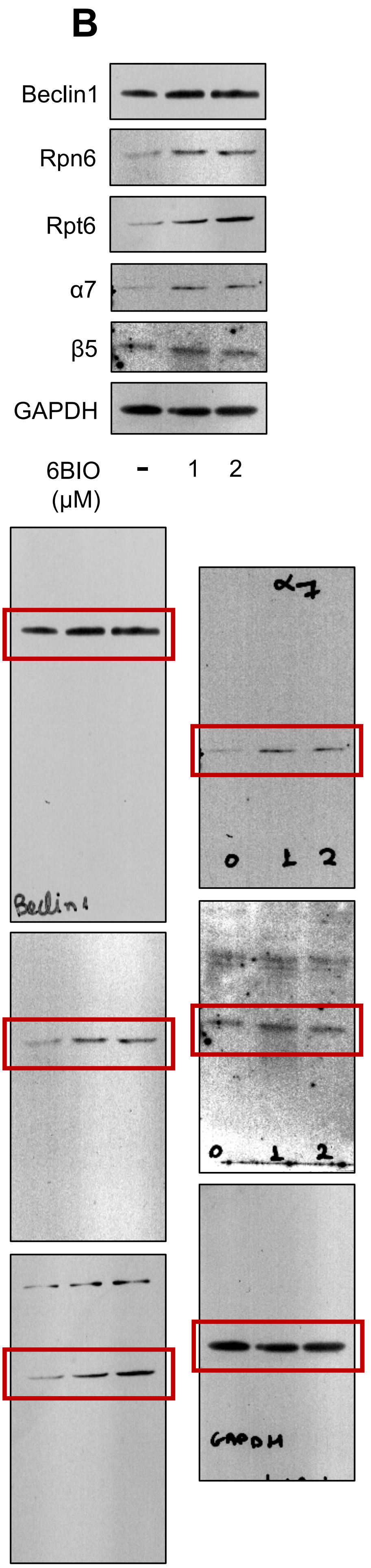

Sklirou et al. Figure 2

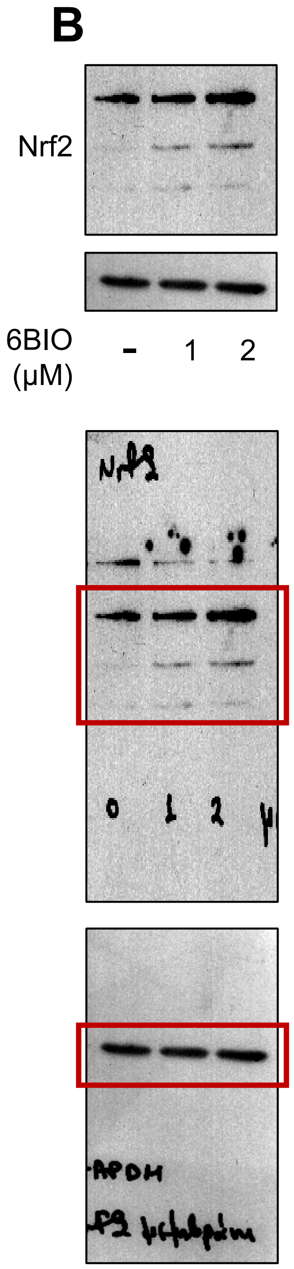

Sklirou et al. Figure 3

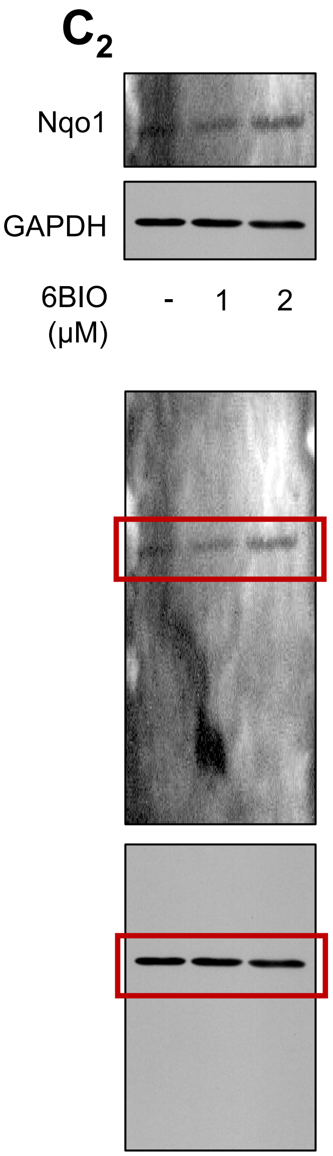

Sklirou et al. Figure 3

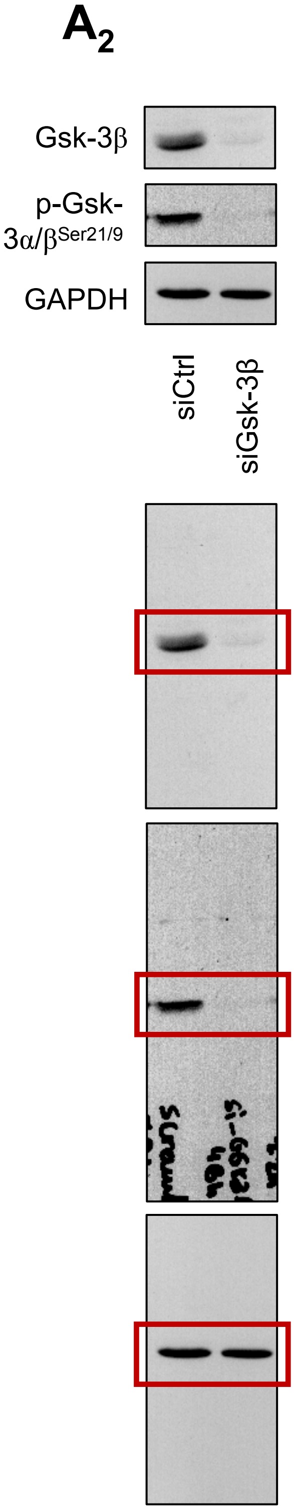

Sklirou et al.
Figure 4

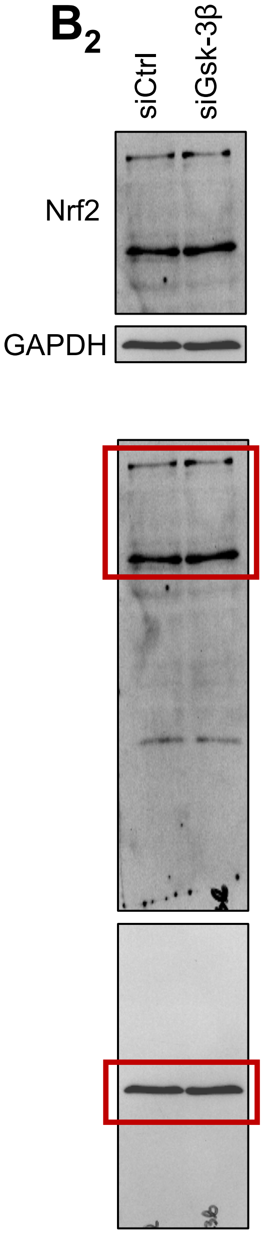

Sklirou et al.
Figure 4

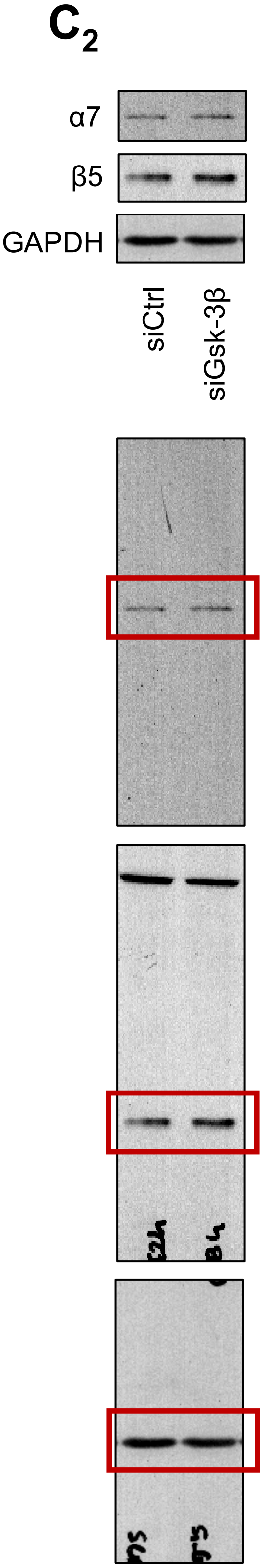

Sklirou et al.
Figure 4

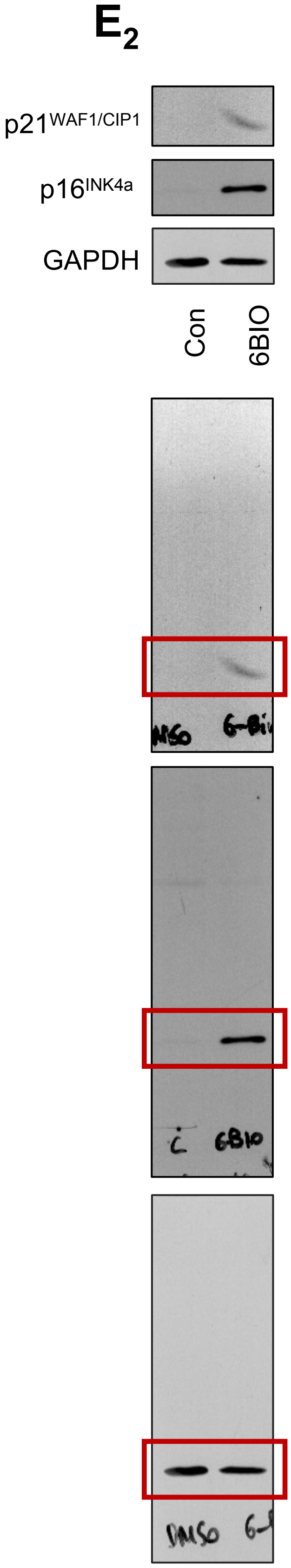

Sklirou et al.
Figure 5

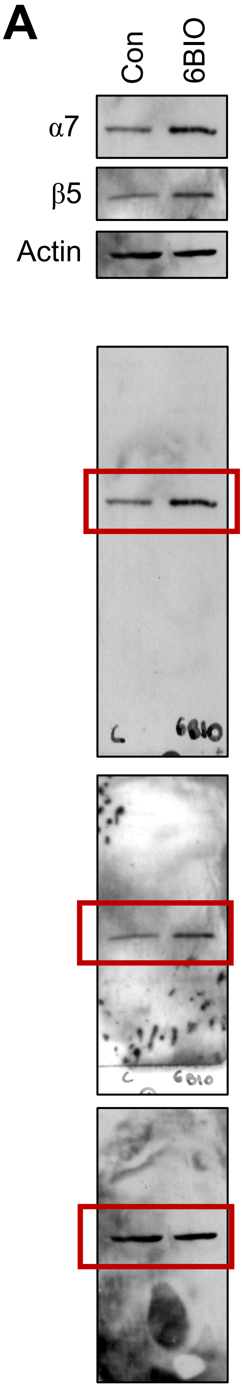

Sklirou et al.
Figure 6

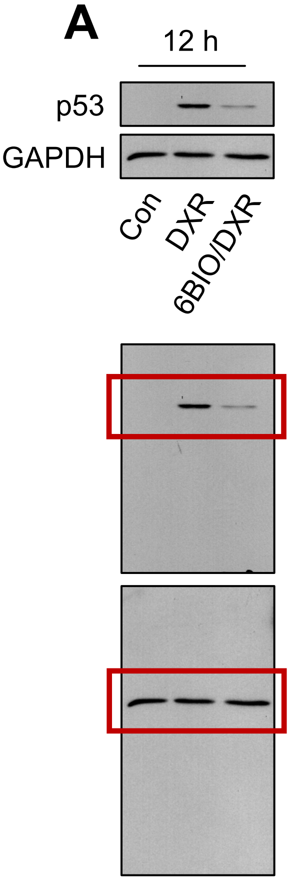

Sklirou et al.
Suppl. Fig. S2

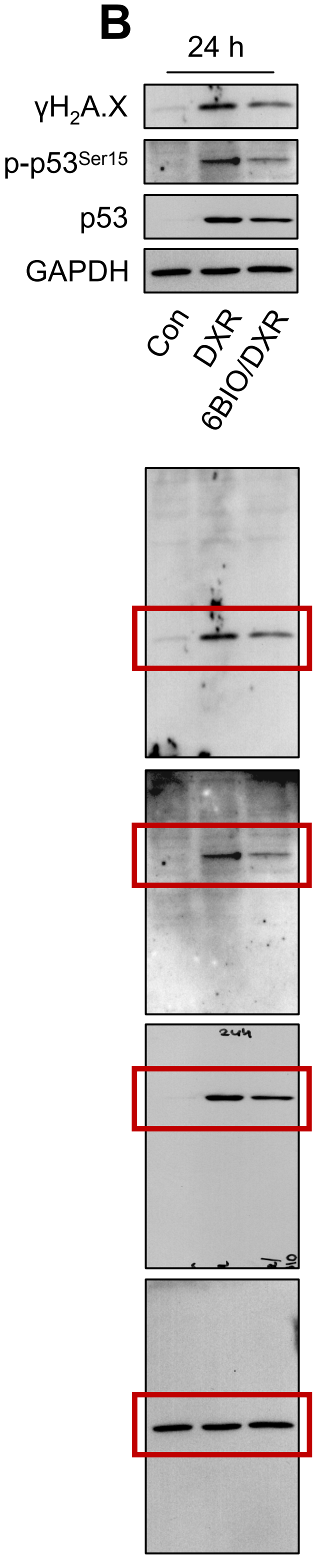

Sklirou et al.
Suppl. Fig. S2

B

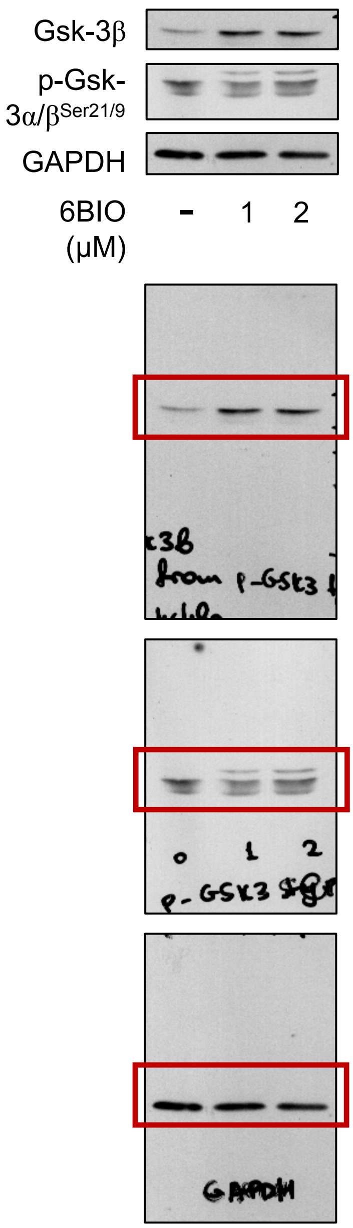

Sklirou et al.
Suppl. Fig. S3

B

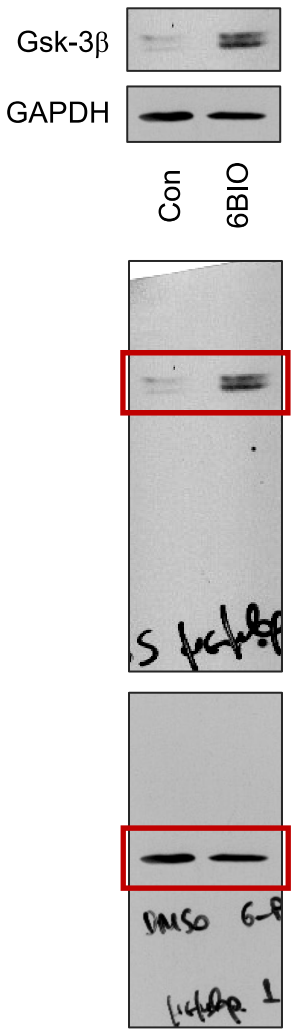

Sklirou et al.
Suppl. Fig. S5
